# A quantitative wildfire risk assessment using a modular approach of geostatistical clustering and regionally distinct valuations of assets—A case study in Oregon

**DOI:** 10.1371/journal.pone.0264826

**Published:** 2022-03-08

**Authors:** Andres Schmidt, Daniel Leavell, John Punches, Marco A. Rocha Ibarra, James S. Kagan, Megan Creutzburg, Myrica McCune, Janine Salwasser, Cara Walter, Carrie Berger

**Affiliations:** 1 Department of Fisheries, Wildlife and Conservation Sciences, Oregon State University, Corvallis, Oregon, United States of America; 2 Department of Forest Engineering, Resources & Management, Oregon State University, Corvallis, Oregon, United States of America; 3 Department of Forest Ecosystems and Society, Oregon State University, Corvallis, Oregon, United States of America; 4 Institute for Natural Resources, Oregon State University, Corvallis, Oregon, United States of America; 5 Department of Biological & Ecological Engineering, Oregon State University, Corvallis, Oregon, United States of America; Zoological Survey of India, INDIA

## Abstract

The intensity and scale of wildfires has increased throughout the Pacific Northwest in recent decades, especially within the last decade, destroying vast amounts of valuable resources and assets. This trend is predicted to remain or even magnify due climate change, growing population, increased housing density. Furthermore, the associated stress of prolonged droughts and change in land cover/land use puts more population at risk. We present results of a multi-phase Extension Fire Program Initiative combining fire model results based on worst-case meteorological conditions recorded at 50 weather stations across Oregon with spatially distinct valuations of resources and assets based on regional ecological and socio-economic conditions. Our study focuses on six different Fire Service Areas covering the state of Oregon. We used a geostatistical approach to find weather stations that provide worst-case meteorological input data on record for representative sub-domains. The results provide regionally distinct assessments of potential value loss by wildfire and show that, depending on the region, 12% to 52% of the highest relative risk areas are on private land. This underscores the need to unite strategies and efforts on the landscape scale by including different landowners, managers, and stakeholders of public land and private land efficiently address wildfire damage protection and mitigation. Our risk assessments closely agreed with risks identified during landscape-scale ground projects.

## 1. Introduction

Increasing global temperatures and alterations in local or regional weather patterns have lengthened fire seasons, reduced moisture content in fuel beds, and induced vegetative changes [1–3]. Simultaneously, human populations and their activities have expanded into forests and other flammable environments, increasing the probability of human-caused ignitions and the potential magnitude of loss to infrastructure, homes, businesses, and human life [4–6].

These trends have led to increased risk and impact of wildfires throughout the Pacific Northwest and the State of Oregon [7, 8]. The Eagle Creek Fire in 2017, for instance, burned 200 km^2^ along a major scenic highway in the Columbia Basin in northern Oregon. The fire came close to a main watershed of the city of Portland, forced hundreds to evacuate, and funneled smoke and ash into the city. The wildfire season of 2020 was the most destructive in recent history in the Pacific Northwest [9]– 2027 fires burned over 4900 km^2^ in Oregon, including several towns and destroying more than 3000 buildings in the western part of the State [10].

In the western United States, there is growing recognition that large fires are not only an issue of climate, but they are also heavily influenced or amplified by past (and current) management practices [11]. The region’s timber harvest, public lands grazing, and wildfire suppression practices all too often focused on short-term objectives. This has resulted in altered forest and range conditions [12, 13] characterized by increased homogeneity of vegetation, shifts in species composition toward those less resistant to fire and more likely to serve as ladder fuels, and increased stand density. These characteristics have led to elevated risks of intense wildfire that can transmit across large landscapes. The probability and consequence of wildfire risk varies both spatially and temporally and is heavily influenced by native fire regimes, extent of deviation from historic vegetative conditions, and the extent of human occupation and distribution of valued infrastructure.

The challenge of wildfire risk reduction is compounded by a lack of resources—U.S. land management agencies suffer from insufficient funding and/or inadequate contractor or internal capacity to address all needs simultaneously [14, 15]. This is true of non-industrial private landowners as well. As such, wildfire risk reduction efforts must be strategically implemented to address highest priorities, with the recognition that not all areas will be feasible for direct treatment. Finally, the need for involvement of individual private landowners and a necessity of broad social understanding and license for action means wildfire risk reduction is not just a management issue—it is also an education and outreach issue and an opportunity appropriate for the Cooperative Extension Program.

In 2018, the Oregon legislature supported the need for holistic efforts to address wildfire risk and approved funding to create the Oregon State University Extension Service Fire Program. The Extension Fire Program was established to advance existing collaborative wildfire risk reduction efforts, help establish new efforts where needed, and increase the rate and reach of on-the-ground wildfire risk reduction treatments on both publicly- and privately-owned or managed land. The Extension Fire Program was specifically tasked with conducting a detailed assessment of fire risk for Oregon to help prioritize efforts. The assessment was to inform strategic placement of six field-based regional fire specialists authorized in the Program’s establishment documentation to fulfill the following: 1. Best serve landscapes and communities with similar needs; 2. Provide initial, regional assessments facilitating development or progress of collaborative networks; 3. Allow for adaptation to local values at a variety of spatial scales, reflective of differences in what (and to what extent) communities value their resources at risk; and 4. Facilitate repeated landscape-scale, cross-boundary assessments that will reveal changing patterns of risk as management treatments or natural disturbances occur.

Various fire risk assessments have recently been conducted. They are based on the combination of a) valuations of assets often generalized across several states, and b) fire model results representing long-term averages of wildfire probability, intensity, and behavior. [16, 17]. The objective of the Extension Fire Program project (which this study serves) is to provide finer-scale relative risk assessments within Oregon to educate communities, agencies, and entities across the State about wildfire hazards and potential impacts on values at risk. Accomplishing this required the development of a method based on modeling fire probability and severity which additionally accounted for spatially distinct valuations of assets and resources as well as some regionally adjusted fire response functions—rather than valuations generalized at statewide or larger scales.

This study introduces the scientific methods used and presents results of the first two phases of the Extension Fire Program in sufficient detail to allow planners, managers, and wildfire specialists to utilize the approach to inform their long-term wildfire risk mitigation efforts at the landscape scale. The objective of this study is to describe the specific approaches of our risk assessment framework in detail and provide a tool tailored to the needs of the OSU Extension Service Fire Program and its collaborators.

Firstly, we describe a data-driven delineation of six distinct areas in the state of Oregon to identify spatially continuous areas that each exhibit maximized homogeneity of climate, vegetation, topography, drought effects, values at risk to hazards, and historic occurrences of wildfires for regionally specific risk assessments.

Secondly, a data-driven selection of meteorological stations most representative of domains for fire model runs is described starting with 163 meteorological stations available in our study area. A reproducible statistic sprawl clustering approach based on physical landscape properties, climate parameters, and data pertaining to fuel type is presented and applied.

Thirdly, we present spatially distinct and intentionally non-universal asset and resource valuations for the example regions, for which the risk to selected resources and assets was calculated. The application of the framework presented is intended to be hierarchical in scale—tiering from the regional risk assessment efforts to ultimately be applied at the site-specific stand scale and thus offer a tool that can be easily customized for localized inputs for fire behavior as well as values at risk.

## 2. Materials and methods

**Fig 1:**
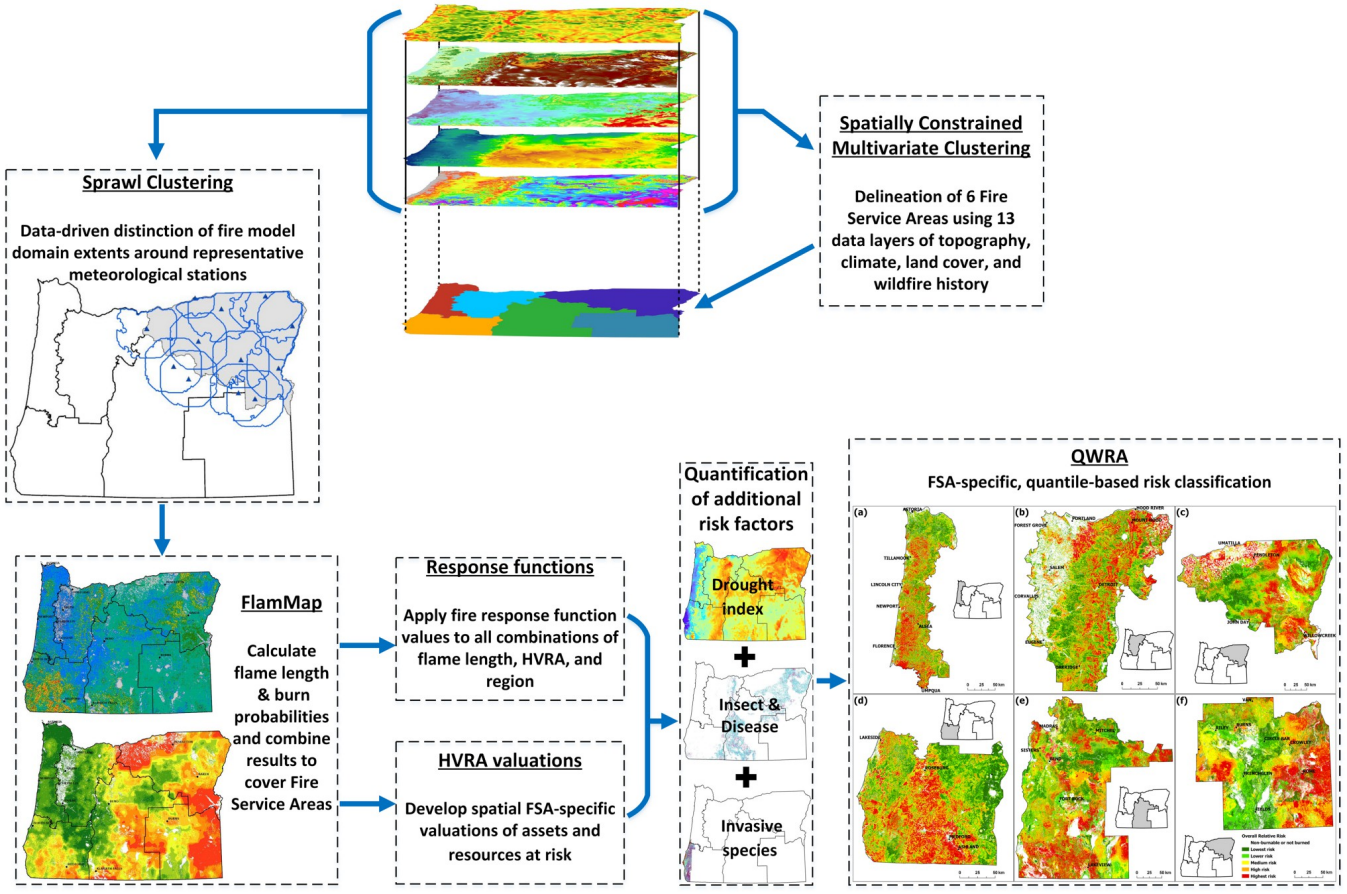
Schematic overview of the processes conducted for the risk assessment.

The Extension Fire Program Relative Risk Assessment (EFPRRA) modeling process involved a series of steps. These included spatial clustering to identify potential “service areas” for the Fire Program’s regional fire specialists, fire modeling augmented by geostatistical selection of meteorological stations, quantification of additional disturbance mechanisms that would be likely to influence fire severity, identification and valuation of regionally relevant resources and assets, and, finally, integration of these components to quantify overall relative risk to regional resources and assets. Each modeling aspect is described in this section. A schematic of the processes is shown in Fig 1.

### 2.1 Delineation of Fire Service Areas

Phase 1 of this project delineated Fire Service Areas (FSAs), within Oregon. These constitute the geographic assignments of the Fire Program’s six regional fire specialists, as supported by the Oregon Legislature. Spatially constrained multivariate clustering (SCMC) [18] was utilized to identify spatially continuous clusters that maximized internal homogeneity of climate, vegetation, topography, drought effects, values at risk to hazards, and frequency of wildfires. A total of 13 variables, including 30-year normal values of PRISM climate parameters [19], topographic parameters, land cover type information from the U.S. National Land Cover Database [20, 21], and historic fire frequencies derived from the Monitoring Trends in Burn Severity (MTBS) database [22] were considered in the clustering procedure. (Table A in S1 Text).

All 13 variables were spatially aggregated to 900 m x 900 m resolution grids covering Oregon. This medium resolution captured the spatial variability in sufficient detail to divide the state into regions while accounting for the varying spatial resolution of the input datasets. One thousand permutations of the SCMC algorithm were used to calculate cluster assignment probabilities for each grid cell. Davies-Bouldin validity indices [23] were calculated to assure that the imposed number of six clusters did not violate the inherent topology of the data. The indices suggested that two or three clusters would most denote regions of similarity for our variables of interest, but that six clusters (reflecting the number of positions funded by the Oregon legislature) was an acceptable constraint with the corresponding validity index being in the 50th percentile of all calculated indices for the range of 2 to 20 clusters.

While the primary purpose of Phase 1 was to delineate areas with similar wildfire hazard characteristics, the FSA boundary lines also needed to serve as boundaries for the areas of responsibility for the Extension Fire Program regional fire specialists. To facilitate manageability, the original cluster lines were adjusted to existing political/administrative boundaries such as city limits, county lines, or natural barriers (such as rivers or mountain ranges). Attempts were made to shift boundaries only within areas not clearly aligned with a cluster (i.e., areas with clustering probabilities less than 50%), but in a few cases the imposed geopolitical boundaries necessitated less than ideal alignment with clusters.

### 2.2 Modeling fire hazard

Phase 2 described, characterized, and quantified wildfire-related hazards within each FSA. It utilized fire behavior modeling, burn probability calculations, and regionally specific valuations of resources and assets at risk, as illustrated in Fig 1. *FlamMap* 6.0 [24], accessed through the Interagency Fuel Treatment Decision Support System (IFTDSS) online front end, was used to model burn probability and flame length binned into six fire intensity levels at a 30 m x 30 m spatial resolution. IFTDSS provided a convenient online front-end to select geographic areas and denote landcover, fuel moisture values, and other parameters required for the *FlamMap* model runs, and to visualize the results [25]. *FireFamilyPlus* (ver. 4.2) [26] was used to predict the 1-hour, 10-hour, and 100-hour, dead and live fuel moisture contents needed for the model runs. A random ignition algorithm was applied in combination with the crown fire model [27]. Modeled burn probabilities were used unaltered for further analysis without calibrations to historical burn probabilities, to avoid masking implications of weather modeling.

Meteorological data has a large effect on the results and accuracy of fire models [28, 29]. Our model utilized worst-case meteorological data instead of long-term average conditions to parameterize the fire model runs. This was done to account for the increasing frequency and severity of droughts and projected increase in air temperature in the Pacific Northwest and thereby considering highest risk fire weather conditions as recorded by the stations for our risk analyses. Moreover, if mean-based parameterizations are used, fire hazard can be potentially underestimated for low frequency fire regimes as found in the coastal region of Oregon where low-probability, high-consequence wildfires have recently destroyed significant amounts of assets and resources [30].

One invaluable and frequently used source of meteorological data for fire modeling is the Remote Automatic Weather Stations (RAWS) network, which provides data from weather stations across the conterminous United states [31]. Each RAWS station’s time series were analyzed and the five-day periods that were both hottest and driest were utilized as input to our fire behavior model. This process was based on the hourly time series of relative humidity and air temperature at each selected RAWS site using a 24-hour moving average filter to avoid selection of data based on short peaks or outliers in the time series. The mode of the wind direction and maximum wind speed during that period were also identified and utilized.

The LANDFIRE 2016 REMAP surface dataset was utilized [32, 33] for this study. A limitation of this dataset is that sections of built-up areas containing numbers of structures that can be close to burnable vegetation were classified as non-burnable in the fuel model layer. This also applied to some sparsely built-up areas where structures and wildland intermixed within Wildland-Urban Interface (WUI) areas and outskirts of towns and cities. Unfortunately, at the time this study was conducted, no wildfire model was available to the authors that would have incorporated buildings and structures in the simulation process.

To include these potentially fire prone areas, this assessment utilized an approach as presented by a previous assessment specifically applied to communities [17]. Grid cells in areas around towns and cities, to which LANDFIRE assigned no value for burn probability and flame length probability, were filled by spatially extrapolating the fire model results from neighboring cells. During post-processing, two consecutive moving window filter steps (using a 3 x 3 grid cells filter width) were applied to calculate burn probability and flame length probability values in those areas originally deemed non-burnable by the fire model. Grid cells with surface water were re-assigned to zero burn probability in cases where those areas were included in the moving window width. Fire model results remained unchanged in all other grid cells.

#### 2.2.1 Geostatistical selection of meteorological stations

Choosing the most applicable weather station(s) can be challenging, as neighboring stations often exhibit differing patterns in wind fields and other meteorological data. In regions with heterogeneous terrain, topography and changing land cover influence a station’s meteorology and climatology on a local scale. Modeling that simply relies upon nearest stations may not be the most appropriate. In practice, fire modeling efforts occasionally rely upon local knowledge to identify the most suitable weather station to draw upon, but such expertise is not available in every region and is prone to being bias from personal perception, potentially compromising the reproducibility of data selection and corresponding results.

This assessment utilized a solely data-driven approach to identify RAWS stations representative of climate and landscape characteristics for specific areas around stations. The 163 RAWS stations available in our study area were each assessed using the 13 variables utilized in Phase 1 in order to determine the spatial extent to which each meteorological station represented areas with similar climate, topography, land cover, and historical fire frequencies. The resulting polygons were then used as selected model sub-domains within which the fire behavior model runs were conducted.

An initial circular area with a radius of 2 km (12.6 km^2^) was assigned around each weather station’s location, and the averages and standard deviations of each of the 13 Phase 1 descriptive variables calculated. At this proximity the respective climate, land cover, and burn history, as well as the measured meteorological data of the RAWS station were assumed to be representative of the area surrounding that station. The process was then repeated with the radii extended by 2 km, and the increase of the variance calculated for each variable in each 900 m x 900 m grid within each cluster area.

With each iteration, the area assigned to that station was extended to adjacent grid cells only when both of two statistical constraints were met: 1) the maximum variance among all clustered variables increased by no more than 5% within respective grid cells, and 2) with all 13 variables considered simultaneously, the clustered variables’ centroid position remained stable within the 13-dimensional vector space. If the normalized Euclidean distance from any variable’s average compared to the normalized position of the centroid during the previous step increased by no more than 5%, the corresponding grid cells in that direction were added to the area attributed to that RAWS station. The sprawl clustering process resulted in polygons of varying shapes that reflected areas best represented by each station. Model sub-domains around the RAWS were manually extended when the limited availability of stations would otherwise have led to blank areas in our model domain.

Finally, the number of RAWS stations utilized in the assessment was reduced by eliminating stations whose areas were overlain by other equally or more representative stations. Based on maximum spatial coverage, 50 sub-domains and the weather stations representative of those areas were selected to cover all six FSAs. The statistical boundary values used were selected empirically and signify the best compromise between a suitably large extent of the model sub-domains and homogeneity within the model sub-domains in terms of climate conditions, landcover, and topography. The 30 m x 30 m results of all sub-domains were then merged to fully cover every FSA. The gridded sub-domain results for burn probability and flame length probabilities (six flame length classes) were merged, using the average for areas where grid cells of different model sub-domains overlapped. To account for unavoidable seams where some fire model sub-domains overlapped results of other, adjacent fire model sub-domains, a moving window filter was applied with a filter width of 10 grid cells. The filtered values were then rescaled to the original range of the results to avoid an alteration of the actual probabilities due to the low-pass filter procedure, while at the same time producing more realistic spatial transitions of the burn probabilities.

#### 2.2.2 Quantification of additional disturbance hazards

The EFPRRA incorporated three hazard factors not considered by the fire behavior model, but which potentially increase fire susceptibility and associated losses to values and resources at risk in certain areas. These additional hazard factors addressed long-term climate drought conditions; insects or diseases affecting vegetation (and, hence, fuel composition, condition, and potential to increase wildland fire intensity and severity); and the abundance of European gorse (*Ulex europaeus*, and invasive and highly flammable species). While this list of additional hazard factors was not intended to be complete (further research would be needed for more accurate quantifications of specific hazards as part of fire models and fuel models), the approach allowed for a quantitative consideration of factors with potential to increase fire probability, intensity, severity, and negative consequences [34–36].

The climatic trend we measured and utilized as a model input represented the increased probability of the wildland fire hazard resulting in higher negative consequences to values at risk. Stronger and prolonged droughts as observed in our study area, and that are expected to occur in the near future, exacerbate this potential and impact [8, 37–39]. The values of drought, insect and disease, and flammable shrubs were added to the Integrated Additional Risks Factor (IARF) to quantify the associated increase of wildfire susceptibility of the potential fuel.

Long-term drought was assessed using the Self-Calibrated Palmer Drought Severity Index (SCPDSI), which is locally calibrated to facilitate spatial comparability [40].

SCPDSI data were available as monthly gridded values with a spatial resolution of 2.5 arc minutes, roughly corresponding to 4 km x 4 km in our study area [41]. For each grid cell, average indices were calculated representing dry conditions during the Oregon peak fire season (July—September) over the last six years. This alleviated effects of year-to-year variability while capturing changing climate trends and correlated fuel conditions [7] during recent years. Since the SCPDSI denotes dry conditions as negative values, grid cells with positive values were omitted and negative values were multiplied by -1 (to make them positive), then scaled from 1 to 1.25. Thus, presence of long-term drought within a grid cell could enhance that area’s risk rating by up to 25%.

The additional hazard potential imposed by effects of insects and diseases on the vegetation (as fuel) was incorporated using a long-term plot dataset provided by the USDA Forest Service through the 2018 Update of the National Insect and Disease Composite Risk Map [42]. Using the 2018 dataset update, only plots where conditions exhibited a remaining risk through insects or diseases were considered exposed to additive fire risk in our assessment.

Invasive vegetation can also have strong effects on wildfire in affected areas due to altered fuel composition and potential to burn [43, 44]. While a variety of invasive plant species can be found in Oregon, the EFPRRA focused on European gorse as an exemplary, highly flammable, and regionally important species. Found along the Oregon coast in portions of FSAs 1 and 4, it increases the fire hazard [45] in areas otherwise considered of relatively low fire hazard [16, 17, 46]. Occurrence probability maps based on field samples [47] and a random forest model [48, 49] were used to quantify additional potential risk at grid cells where gorse occurrence was classified as very likely or highly likely (with α > 0.5 indicating high confidence values for the model predictions). Where present, an additional risk factor of 0.25 was added to the grid cell.

The values of the three additional risk factors were combined to derive an integrated additional risks factor (IARF), to which drought contributed 1.0 to 1.25, and presence of insects/disease and/or gorse contributed a maximum of 0.25. Thus, the IARF could increase fire risk ratings by a maximum of 50% (see Eq 4).

#### 2.2.3 Resources and assets at risk

Twenty-one Highly Valued Resources and Assets (HVRA) were considered in the EFPRRA. Note that the set of HVRAs used makes no claim to be complete yet incorporates a wide range of assets and resources potentially at risk through wildfire in the study area. Furthermore, we introduce distinct and intentionally non-universal asset and resource valuations as used for the Extension Service Fire Program within Oregon for which the risk to selected resources and assets was calculated. The HVRA considered in this study and conducted on the FSA scale included measures such as population density, WUI, several building/structure categories, infrastructure considerations such as power and communication lines, recreation resources, agriculture, timber, wildlife and endangered species habitat, and water-related resources/considerations.

The values at risk side of risk assessments were developed by collaborating with the Oregon State University-based Institute for Natural Resources (INR). INR specializes in the synthesis of expertise, research findings, data, tools, and information. We worked cooperatively with INR to identify and valuate our initial values at risk. Each HVRA was identified as a resource or asset known to be of value to Oregonians for which georeferenced data was available at 30 m x 30 m (or better) resolution (Table 1).

**Table 1:** Scaled valuations for HVRA subcategories by FSA.

HVRA main category	HVRA sub-category	Scaled valuation					
		FSA 1	FSA 2	FSA 3	FSA 4	FSA 5	FSA 6
People	Population density	Grid cell explicit (range: 0–9)					
	Wildland urban interface	5	5	5	5	5	5
Buildings	Building density	Grid cell explicit (range: 0–9)					
	Historic buildings	6	6	6	6	6	6
	Fire response buildings	7	7	8	7	8	9
	Buildings with vulnerable people	9	9	9	9	9	9
Infrastructure	Communication infrastructure	7	7	7	7	7	7
	Power infrastructure	Grid cell explicit (range: 0–9)					
	Transportation infrastructure	6	6	6	6	6	6
	Recreation sites	2	3	2	2	2	2
	Sawmills	4	3	5	4	5	5
Agriculture	Agriculture	Grid cell explicit (range: 0–9)					
Timber	Timber volume in harvestable areas	Grid cell explicit (range: 0–9)					
Habitat	Habitat	Grid cell explicit (range: 0–9)					
Species	Threatened, endangered and at-risk	Grid cell explicit (range: 0–9)					
	Critical habitat for key species	Grid cell explicit (range: 0–9)					
	Big game	Grid cell explicit (range: 0–9)					
	Salmon	3	3	3	3	3	3
Water	Drinking water from groundwater	5	6	5	6	6	5
	Drinking water from surface water	4	4	4	4	4	4
	Scenic waterways	3	3	3	3	3	3

Note that valuations by FSA are only provided for presence-absence datasets. Continuous datasets are scaled from 0 to 9 for each grid cell specifically and regional differences across the state are incorporated into the respective HVRA raster datasets.

HVRAs with continuous datasets were linearized to a 10-class integer scale ranging from 0 (no value) to 9 (highest value). HVRA datasets denoting presence or absence of a resource (by grid cell) were mapped as binary values (0 if absent, weighted value if present) and valuations assigned from lookup matrices during processing. All valuations were gridded and aggregated to 30 m x 30 m spatial resolution for each subcategory.

Whereas risk assessment models typically assign statewide values to resources and assets, EFPRRA fine-tuned valuations regionally (at the FSA level) in recognition of the widely different socio-economic conditions and ecosystem services provided in different parts of Oregon [50, 51]. For instance, a hospital or fire station in a rural area (where these assets are scarce) may merit a higher value assignment than those same resources in more populated areas that benefit from greater numbers of the assets. The valuations were scaled relatively within each HVRA main category, but not across main categories. For example, we weighted the value of historic buildings in relative to fire response buildings, but we do not suggest that a mapped value of 5 in the people category is equivalent to a mapped value of 5 in the timber category. Additionally, HVRA weights and values were computed independently for each FSA to enable each region to customize how it values its resources and assets. As such, a mapped value of 5 in the timber category in FSA 1 is not necessarily the equivalent of that same value in FSA 2. Therefore, weights and values calculated are relative to their FSAs rather than being equal throughout the state. Table 1 provides valuations by category and FSA. Descriptions of HVRA main categories follow.

The People HVRA main category assigned values for population density and WUI classification. Population density estimates were obtained from the 2018 Land Scan USA Population Database provided by U.S. Department of Homeland Security through the Homeland Infrastructure Foundation-Level Data database (HIFLD) [52, 53].

Separate day and night gridded datasets were averaged into a single raster and values reclassified based on a quantile classification. Population density was structured as a continuous dataset with distinct values for each 30 x 30 m grid cell and therefore was not weighted by FSA.

WUI areas were assigned a valuation of 5 across all FSAs. WUI was considered of intermediate importance and no information was available to weight these areas differently in different FSAs. WUI areas were represented as a presence-absence grid, based on a dataset released by the USDA Forest Service for the conterminous United States [54] and updated in May 2020.

The Buildings HVRA included four elements: building density, historic buildings, fire response buildings, and buildings with vulnerable people. The Building Density data layer provided the number of buildings per km^2^ across Oregon, with building locations extracted from the 2018 Microsoft Building Footprint database created from satellite and aerial imagery using the ResNet34 deep neural network [55]. The density values were rescaled to 0–9 using a quantile classification (Table 2).

**Table 2:** Density-based valuation of buildings used for the risk assessment.

Buildings/km^2^	HVRA valuation
0	0
> 0–1	1
> 1–3	2
> 3–5	3
> 5–8	4
> 8–13	5
> 13–21	6
> 21–37	7
> 37–89	8
> 89–1633	9

Historic Buildings in Oregon were identified using the 2014 National Park Service National Register of Historic Places Public Dataset [56]. Historic Buildings were assigned a scaled valuation of 6 across all FSA regions because they are valuable but not considered as valuable as emergency response buildings or buildings housing vulnerable people (Table 1).

Fire Response Buildings (fire departments) values ranged between 7 and 9, due to their importance in responding to wildfire. They were weighted higher in FSA regions 3, 5 and 6 (eastern Oregon) because these regions featured fewer stations covering larger areas. Locations of fire response buildings were obtained from the HIFLD repository [53].

The Buildings with Vulnerable People HVRA incorporated buildings merged from nine separate spatial data sources (S2 Text) for medical facilities, shelters, schools, and nursing homes. Buildings with vulnerable people were assigned the highest valuation (9) across all FSA regions because they contain populations at high risk which would be susceptible to wildfire impacts and difficult to evacuate during an emergency, regardless of their FSA location.

The Infrastructure HVRA main category was divided into five subcategories: communications; power; transportation; recreation; and sawmills (Table 1).

The Communication infrastructure data layer included locations of cellular towers, FM transmission towers, AM transmission towers, microwave service towers, and various land mobile transmission towers across the state from the HIFLD database. Communication infrastructure was given a valuation of 7 across all FSAs due to its importance in coordinating emergency response and informing the public of hazards.

The Power infrastructure HVRA included transmission lines and substations, wind turbines, and power plants. Items in the power infrastructure HVRA dataset were assigned a valuation of 0 where no power infrastructure exists, 3 where transmission lines were located, 6 where wind turbines were located, and a value of 9 where substations or power plants were located. Where multiple power infrastructure facilities were mapped in the same grid cell, the maximum of 9 was used as the final HVRA valuation. Power infrastructure was not weighted by FSA but was grid cell-specific.

Transportation infrastructure was consistently valued at 6 because it is less valuable during emergencies than communication infrastructure, but still important for commerce, evacuation, and emergency response—all of which could be impacted by wildfire.

Recreation sites were assigned a value of 2 in all FSA regions except for FSA 2, where the resource was given a value of 3 in recognition of its recreation sites (near population centers) experiencing greater usage. The recreation sites HVRA data includes ski areas and other recreation sites and facilities. Both datasets were compiled for the Pacific Northwest Quantitative Wildfire Risk Assessment 2018 report [16] and original data layers were obtained by request from the report authors.

Sawmills were considered a valuable element of the infrastructure HVRA main category because of their local importance to the timber industry in Oregon [57]. Sawmills were given ratings between 3 and 5, with highest values in FSA 3, 5, and 6 because of their higher relative contributions to local economies and their sparse distribution in those regions (Table 1).

The Agriculture HVRA included crops of high economic value that would be vulnerable to wildfire. Crops’ economic values were determined based on an annual assessment by the Oregon Department of Agriculture [58]. Each Oregon crop was assigned a relative crop value based on their economic value, their rank as a top-20 agriculture commodity, and their national ranking of agricultural production. *CropScape* data, provided by the National Agricultural Statistics Service [59, 60], was used to quantify the spatial distributions and types of crops cultivated in Oregon for the year 2019. In addition, the spatial distribution of winter wheat and caneberries from the 2017 and 2018 CropScape dataset were included because these crops were not well represented in 2019 (their land areas had been mapped as fallow fields). Crop economic values were rated from 0 (no value) to 3 (highest value). Detailed valuations for all crops considered are provided in the S1 Table.

The category Timber Volume in Harvestable Areas estimated the amount of timber available in forested lands where timber harvesting activities may occur. Timber volume was derived from the 2014 Gradient Nearest Neighbor (GNN) forest structure map [61], which estimated the volume (m^3^/ha) of live trees > = 2.5 cm diameter at breast height (ca. 1.37 m). Areas not likely to provide timber were adjusted using the Protected Areas Database 2.0 [62], which revealed national parks, wilderness areas, research natural areas, nature reserves, and other protected areas. Areas excluded from timber harvest were those determined to have Gap Analysis Program (GAP) [63] Status 1 or 2.

Accordingly, the GNN forest structure layer was clipped to set designated protected areas (GAP status 1 or 2) to a value of 0, indicating no potential for timber harvest. For all other forested grid cells, live tree volume estimates from the GNN were classified into 10 categories (Table 3) on a scale from 0–9 based on quantiles of live tree volume. Classifications were done separately for timber volume on the eastern side (FSA 3, FSA 5, and FSA 6) and western side of the state (FSA 1, FSA 2, and FSA 4) as their relative productivities vary rather dramatically.

**Table 3:** Classification of timber volume for value assignment and fire response allocation.

Timber volume (m^3^/10000 m^2^)		Value Class
East (FSA 3, 5, 6)	West (FSA 1, 2, 4)	
0	0	0
≤ 15	≤ 42	1
≤ 32	≤ 107	2
≤ 54	≤ 178	3
≤ 78	≤ 258	4
≤ 105	≤ 341	5
≤ 135	≤ 437	6
≤ 175	≤ 573	7
≤ 243	≤ 766	8
≤ 1614	≤ 6241	9

The Habitat HVRA assigned values, ranging from 0 to 9, to 77 different habitat types identified in the Oregon Statewide Habitat Map developed by the Oregon Biodiversity Information Center [64, 65]. Details are provided in the S2 Table. Note that, for this assessment, western juniper habitat was divided into “old” juniper and “other” juniper (primarily juniper expanding into historic shrub steppe sites), as old juniper is generally protected during management while other/expanding juniper is targeted for removal. Note also that while the habitat map contains some information about forest structure it does not identify stocking density. As a result, it fails to differentiate between fire-resistant/adapted stands of old ponderosa pine or Douglas-fir and overstocked forests of mixed small and large trees which could have very different economic or societal values.

The Species HVRA main category included four subcategories: Threatened/endangered/at-risk species; critical habitat for key species; big game; and salmon. The threatened, endangered and at-risk species dataset was derived from the Element Occurrence Record (EOR) geodatabase of the Oregon Biodiversity Information Center (OBIC) [66]. It contained over 38,000 records, each identifying an at-risk species population. A total of 1751 species, each occurring in 1 to 5 FSAs, were identified.

Species with a status of “secure” or “probably secure” in Oregon were assigned ratings of 0. Populations of endangered species that could be extirpated by wildfires, such as endangered butterflies or federally listed species occurring in old-growth forests, were attributed a value of 9. Within each grid cell, values for each species present were summed, resulting in grid cell-specific totals ranging from 0 to 72. This range was then reclassified to a 0 to 9 range (Table 4).

**Table 4:** Composite valuations for the species category based on EOR.

Sum of attributes	Classified valuation
0	0
1	1
2	2
3	3
4	4
5	5
6	6
7–8	7
9–10	8
11–72	9

Critical Habitat for Key Species considered mapped habitat for 18 species listed by the US Fish and Wildlife Service. Data was downloaded from the Environmental Conservation Online System of the US Fish and Wildlife Service (https://ecos.fws.gov/ecp/report/table/critical-habitat.html). A recently proposed species, the greater sage grouse (*Centrocercus urophasianus*), was added to the list. Assigned values ranged from 0 to 2 (Table A in S3 Text). Where habitats for species overlapped, scores were summed. For example, forested grid cells with both spotted owl (*Strix occidentalis*) and marbled murrelet (*Brachyramphus marmoratus*) habitat were assigned a value of 4, whereas grid cells with only one of the species scored a 2. The range of summed values had a maximum of 6.

Big Game habitat summarized maps of winter range habitat for deer and for elk in Oregon obtained from the Oregon Department of Fish and Wildlife web database (https://nrimp.dfw.state.or.us/DataClearinghouse). The raster contains values from 0 to 2, with 0 values where no deer or elk winter range habitat was mapped, a value of 1 where there was winter habitat for either deer or elk, and a value of 2 for areas that provided both deer and elk winter habitat. The dataset had a maximum value of 2, recognizing that other species in this HVRA category are more valuable and vulnerable to wildfire.

Salmon habitat identified essential habitat for salmonids from a 2015 layer provided by the Department of State Lands (DSL) and Oregon Department of Fish and Wildlife (ODFW), available online as an ArcGIS service (https://chetco-new.dsl.state.or.us/arcgis/rest/services/Maps/ESH_State/MapServer) but obtained directly from DSL for this project. The map was used in lieu of the National Oceanic and Atmospheric Administration critical habitat maps of federally listed salmon, primarily because the ORBIC EOR already included salmon streams and rivers in Oregon. Additionally, the Essential Salmon Habitat map was specifically defined in Oregon law (OAR 141-102-0030) as representing the state’s significant salmon areas. Salmon were given a rating of 3 across all FSA regions, where present, because as an aquatic species they were inherently less prone to direct wildfire damage, and most salmon species were also captured in the threatened, endangered, and at-risk species subcategory.

The Water main HVRA category consisted of three subcategory layers: drinking water from groundwater, drinking water from surface water, and scenic waterways. Drinking water from groundwater was assigned values of 5 or 6 depending on the FSA (Table 1). Drinking Water from Surface Water was rated as 4 across all FSA regions. Scenic Waterways were considered less vital than drinking water sources and valuated with 3 across all FSA regions.

#### 2.2.4 Fire response functions

Fire response functions quantify the consequential degree of harm (or benefit) to a certain value at risk if exposed to fire with a certain intensity, expressed as six flame length heights. EFPRRA response functions were primarily adopted from the Pacific Northwest Quantitative Wildfire Risk Assessment 2018 [16] but were in some instances refined or added to reflect FSA-specific conditions or expected outcomes. The response functions consist of positive percentage values for beneficial effects and negative values for damaging effects. Each HVRA (Table 1) was represented by a matrix of response function values reflecting six flame length categories (0–0.6 m, 0.6–1.2 m, 1.2–1.8 m, 1.8–2.4 m, 2.4–3.65 m, and >3.65 m). Response functions modified or developed specifically for the EFPRRA are shown in the S3 Table and can be summarized as follows:

- WUI response functions were obtained from [67], averaged across their three WUI types.
- Timber functions were segmented and adjusted to reflect differing conditions for the western (FSAs 1, 2, and 4) and eastern (FSAs 3, 5, and 6) sides of the state.
- HVRA grids related to density values such as housing density, population density, or timber volume were assigned various sets of response functions for different density classes based on quantiles of the distribution of density values.
- HVRA grids that refer to assets and resources with many sub-types, such as agriculture and habitat, were assigned specific fire response values for each type of crop or habitat. For agricultural resources, the response function values applied were based on an earlier, unpublished project study at the Institute of Natural Resources at Oregon State University that examined fire sensitivities of various groups of crops considering general sensitivity and irrigation status. Seventy-three crops cultivated in Oregon were assigned to four different fire sensitivity classes. We adopted that classification and applied a 6 (flame lengths) x 4 (crop fire sensitivity classes) response function array individually on the grid cells to account for flame length and crop type.

#### 2.2.5 Conditional flame length

Conditional flame length (Eq 1) was calculated as a representation of relative risk independent of HVRA considerations. It provides a consolidated estimate of fire intensity, incorporating all six flame length classes and was calculated as the sum overall flame length occurrence probabilities according to:

$$\sum_{i=1}^{6}FLP_{i}\cdot FL_{i},$$ (1)

where *FLP*_*i*_ is the probability of fire with a specific flame length, *FL*_*i*_ is the mid-point of that flame length class, and *i* is the flame length class. An upper limit of 7.6 m was applied when calculating the mid-point of *FL*_6_, the flame length class of 3.65 m and above.

#### 2.2.6 FSA-specific overall relative risk calculations

For each FSA, regionally distinct HVRA valuations, HVRA-specific fire response functions, and the fire behavior model results were combined similarly to the approaches utilized in earlier regional and local fire risk assessments [68–71]. First, risk for each HVRA (within a specific FSA) was calculated as:

$$R_{j}=\sum_{i=1}^{6}FLP_{i}\cdot RF_{ij}\cdot RV_{j}$$ (2)

Where:

*R*_*j*_ = Risk associated with the j^th^ HVRA (*j* = 1, …, 21) within the specific FSA being modeled.

*FLP*_*i*_ = Flame length probability for i^th^ flame length class (*i* = 1, …, 6).

*RF*_*ij*_ = Response function associated with each combination of flame length and HVRA.

*RV*_*j*_ = Valuation of asset/resource for a particular HVRA.

Next, the HVRA-specific risk assessments were summed to arrive at the Relative Risk for each grid cell (again derived individually within each FSA).


$$RR=\sum_{j=1}^{21}R_{j}$$ (3)

Finally, the overall relative risk within each FSA was calculated for each 30 m x 30 m grid cell by multiplying its relative risk, burn probability, and IARF:

ORR=RR⋅BP⋅IARF, (4)

where:

*ORR* = Overall relative risk

*RR* = Relative risk

*BP* = Burn probability

*IARF* = Integrated additional risks factor.

ORR lacks a direct physical meaning or unit. Rather, it comprises a scaling system for relative valuation and offers an integrative measure combining fire probability, fire severity, fire sensitivity of each HVRA, and FSA-specific valuations of HVRAs into one interpretable value, which in turn facilitates a quantitative risk assessment.

To account for regionally specific fire risks and values assigned to assets in differing regions of the state, overall fire risk was modeled individually for each FSA. The five risk categories (ranging from lowest risk to highest risk) were based on quantiles of ORR values of all grid cells calculated for a specific FSA. This led to a classification of the relative integrated risk value that addressed the regionally distinct combination of fire risk driven by burn probability and flame length, as well as the FSAs’ specific values of assets and resources (Eq 4). Hence, a resource potentially affected by fire damage and rated at being at highest risk to hazard in one FSA could be assigned to a different risk class in another FSA. While potentially positive effects of lower severity fires are considered through some of the response function values used for the Timber HVRA (S3 Table) or the Deer & Elk Habitat HVRA, our ORR classification does not include a “positive effect” bin.

Hence, if in summary effects were overall positive for a given grid-cell this would lead to a assignment to the quantile-based lowest ORR class, including grid cells where the effects of a fire would be beneficial for a specific HVRA included in the sum (Eq 3). We decided to use this classification including five risk classes with no beneficial allocation in the final classifications system because a “positive effect” is not considered a risk and therefore is not specifically labeled in our final ORR classification.

## 3. Results

### 3.1 Large-scale outcomes

**Fig 2:**
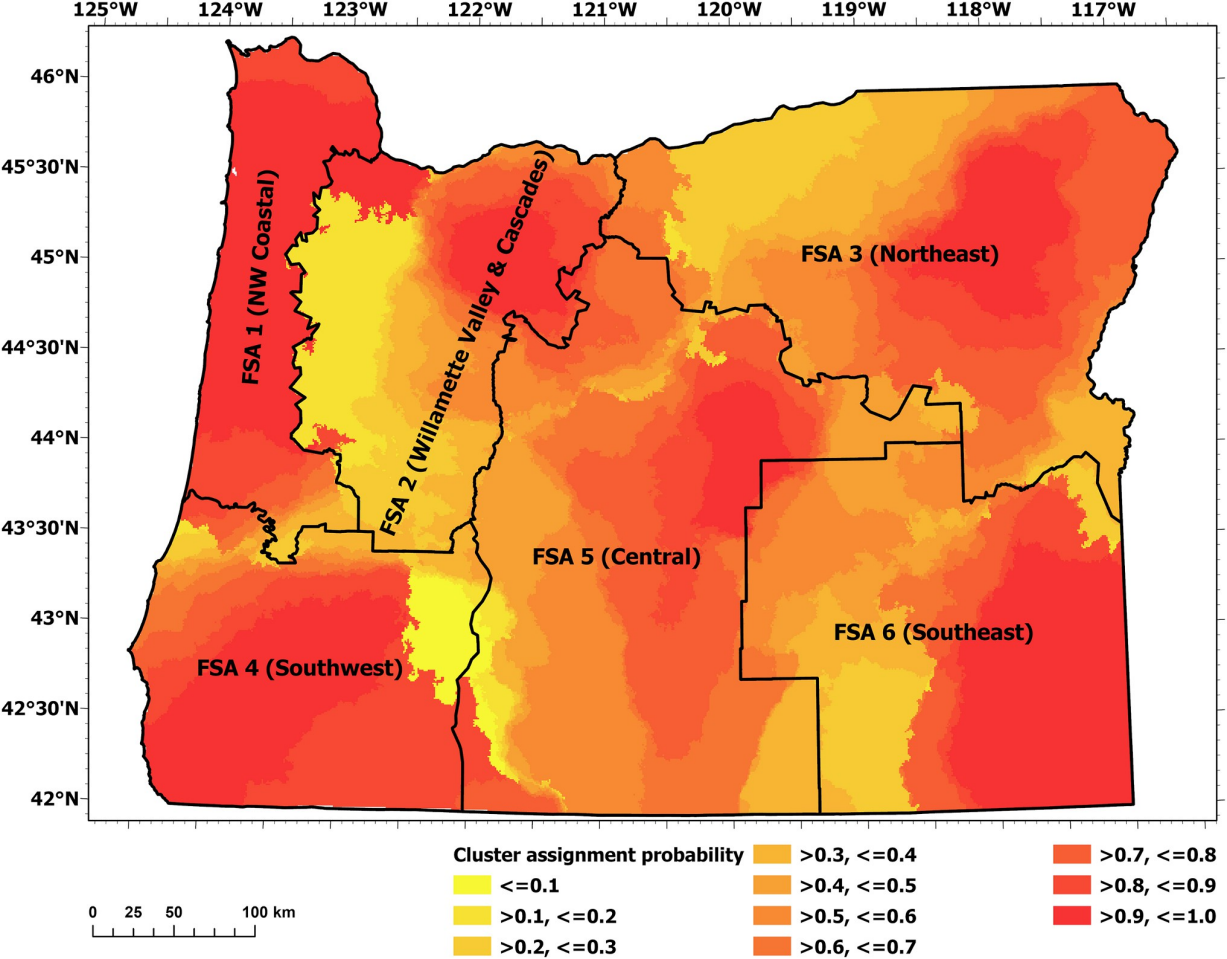
Cluster assignment probabilities of the SCMC procedure including the overlaid final FSA boundaries.

Cluster assignment probabilities were generally high within each individual FSA, and lowest at their margins. The exceptions occurred where FSA boundaries were shifted to align with geopolitical boundaries (typically county lines) or natural boundaries (e.g., rivers or mountain ridges). These regions formed the analysis areas for subsequent modeling. The final FSA delineations and cluster assignment probabilities are shown in Fig 2.

**Fig 3:**
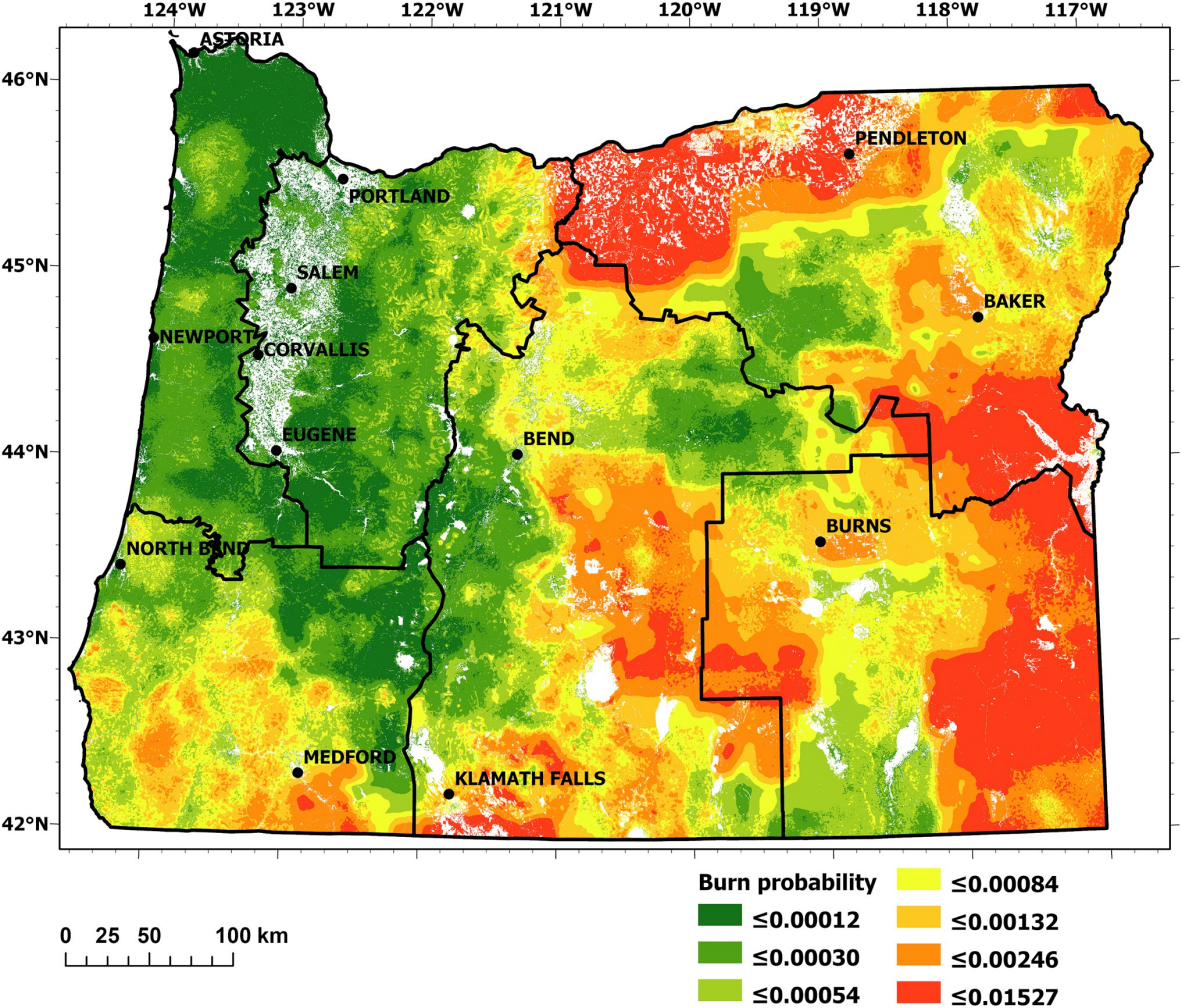
Burn probability for all FSAs using worst-case meteorological data on record during peak fire season from the 50 selected weather stations.

**Fig 4:**
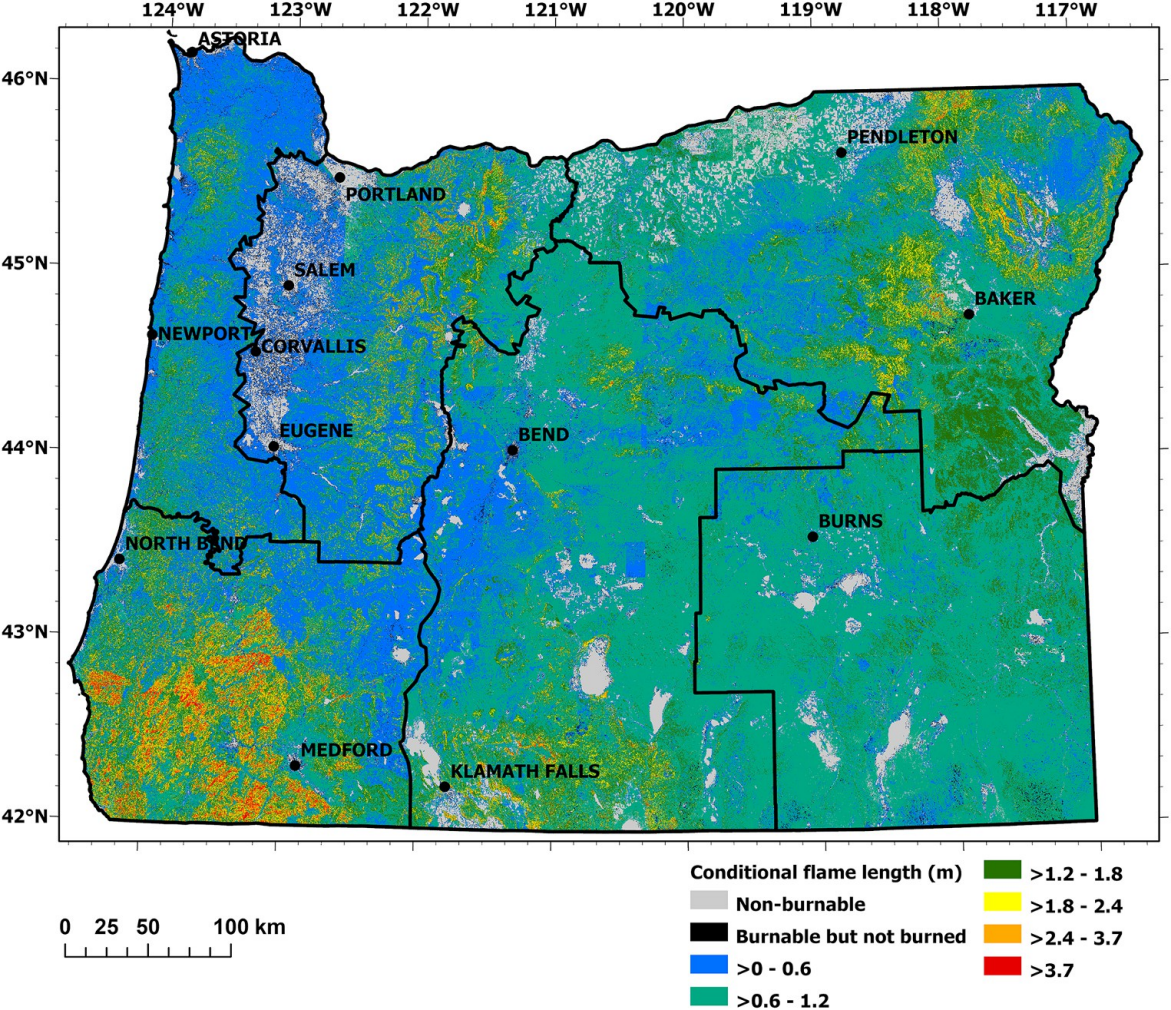
Conditional flame length modeled for each FSA.

Burn probability and conditional flame length are illustrated in Figs 3 and 4, respectively. The modeled results of these large-scale spatial patterns strongly corresponded to climate and vegetation gradients (particularly precipitation) within subregions: the mesic western part dominated by coastal regions; the productive Douglas-fir forests in the Coast Range Mountains and the western flank of the Cascade Mountains; the Willamette Valley; and the dry to semiarid regions east of the Cascade Mountains crest. The spatial patterns, as shown in Figs 3 and 4, largely agree with other the recent burn probability and fire severity modelling efforts conducted at the statewide scale [16, 17, 46]. Due to the difference in fire models employed (FlamMap vs. FSim), and data preparation/post-processing (e.g., our model used unaltered burn probabilities while some other models shifted burn probabilities towards historical fire occurrence), the absolute values of burn probability and flame length differ between among the studies within an expected range. Furthermore, due to the quantile-based scaling of our overall risk classification (section 2.2.6), absolute values of burn probability and flame length probability do not alter risk classifications but rather the relative differences among grid cells within each FSA. Note that a detailed comparison among fire models was beyond this study’s scope.

Most extreme conditional flame lengths were predicted for heavily forested (and thus high fuel load) regions such as the Coast Range and Cascade Mountains. In particular, the densely forested Siskiyou Mountains located in southwestern Oregon (FSA 4) featured dense, mixed conifer forests highly susceptible to intense wildfires—results attributable both to high fuel loads and low fuel moisture due to recurrent summer droughts.

The meteorological data we applied did not consider potential future changes of climate that are expected to further exacerbate conditions for wildfires. Instead, our data represent worst case conditions as already observed and recorded by weather stations, leading to results reflective of conditions likely to recur more frequently in forthcoming years.

**Fig 5:**
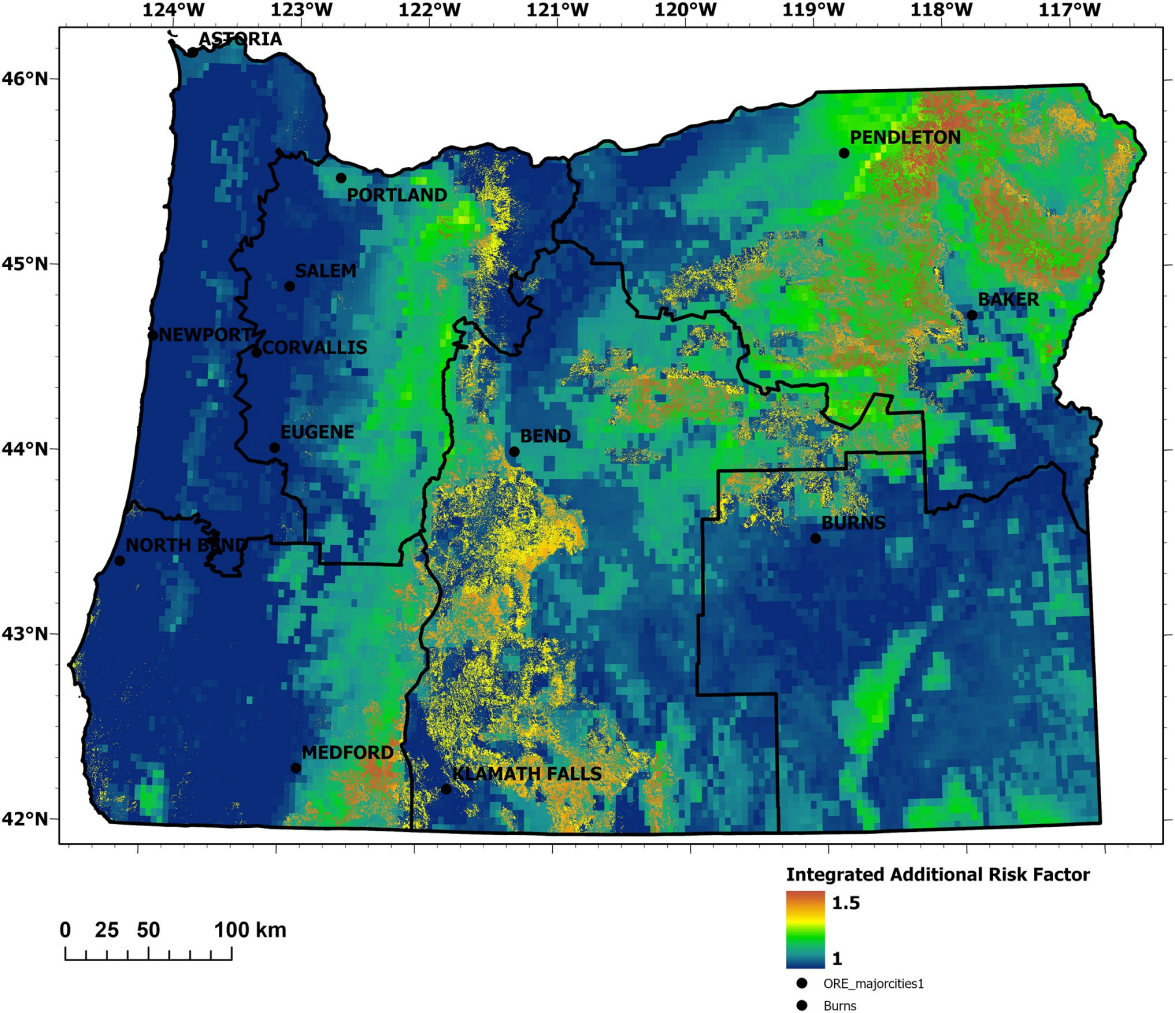
Spatial distribution of the IARF accounting for drought severity during the fire season, insect and disease infestation, and gorse occurrence.

The IARF generally followed delineations of insect and disease issues which are often correlated with long-term drought [72]. The exception is an area along the southern Oregon coast which has been invaded by gorse (Fig 5).

**Fig 6:**
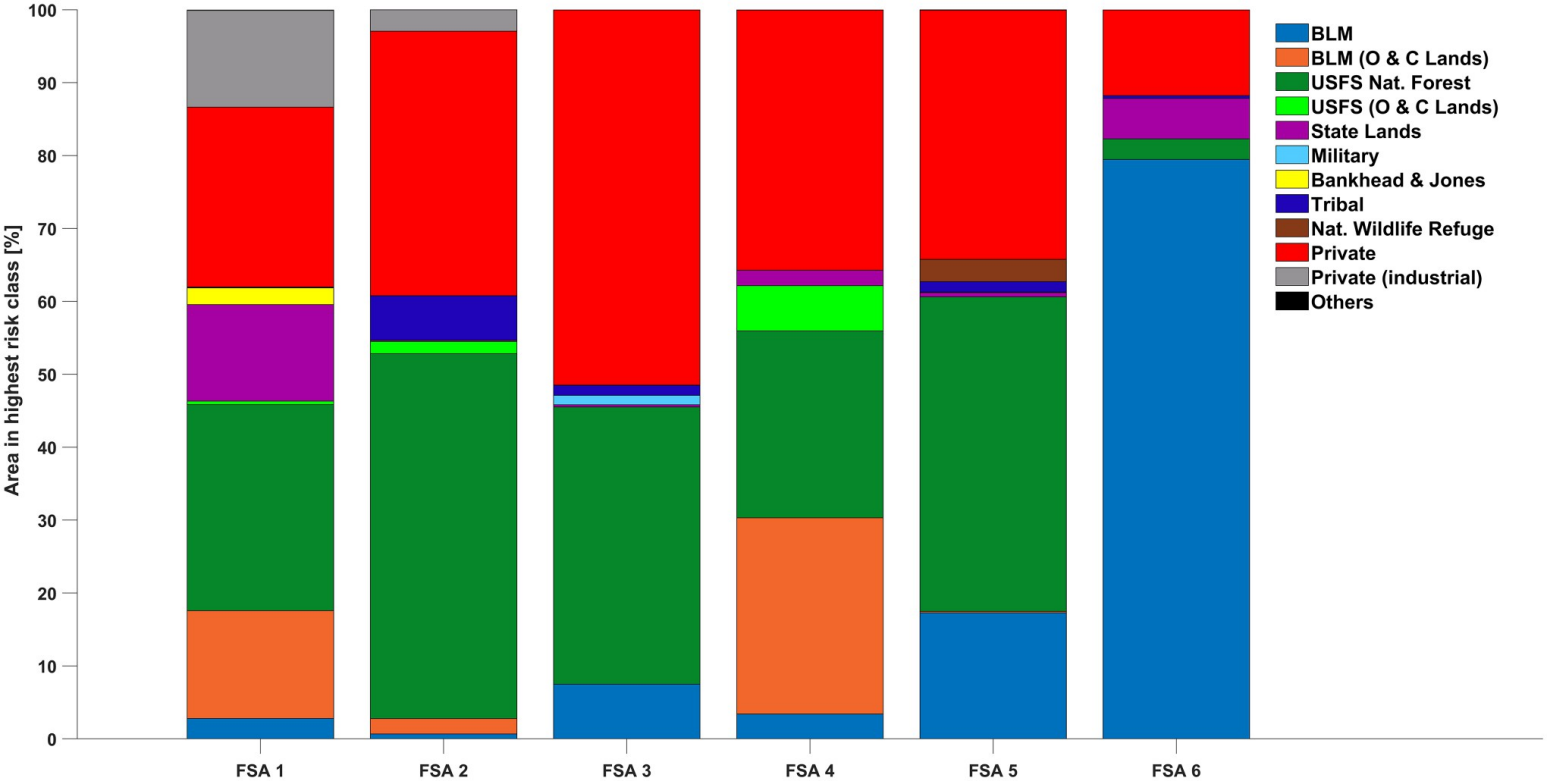
Percentages of land owned and managed by different entities in areas with the highest ORR within each FSA. Entities with less than 1% assigned to the highest risk class in any FSA were summarized as ‘Others’.

A significant proportion of land in Oregon is privately owned, and risk levels associated with private lands varied by FSA. Per Fig 6, the percentage of areas assigned to the highest ORR category ranged from 11.8% (FSA 6) to 51.5% (FSA 3).

**Fig 7:**
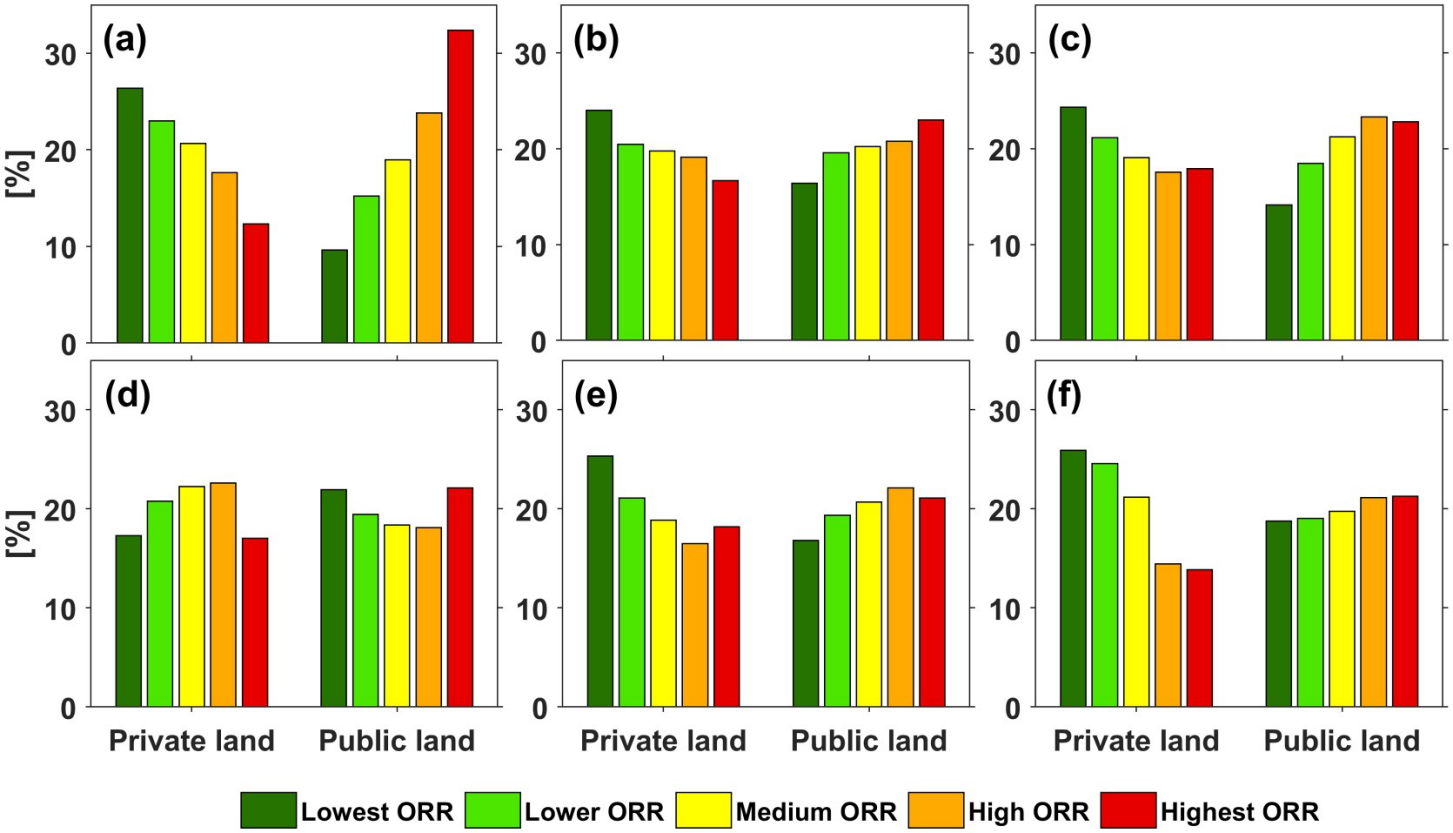
ORR class allocations showing the percent of private and public land area, respectively, assigned to the risk classes in FSA 1 to 6 (panel a to f).

Fig 7 shows the distribution of ORR on private and public land in Oregon. The values show that with exception of FSA 4, the largest percentages of public land are assigned to the high ORR and highest ORR classes whereas the biggest portion of private areas is assigned to the lower and lowest ORR classification which agrees with similar distributions found in recent related studies [17, 30].

A detailed analyses of ORR findings are shown in subsections 3.2.1 to 3.2.6 for each FSA.

### 3.2 FSA-specific outcomes

#### 3.2.1 FSA 1: Northwest coastal

FSA 1 comprises the northwestern coastal region of Oregon and covers an area of 20265 km^2^. The region is characterized by the mesic forests of the Coast Range mountains with high timber values and a sparsely populated coastline with tourist infrastructure of high importance for the regional economy. Its average annual air temperature is 10.6°C. With 2063 mm the area features the highest annual precipitation values among the FSAs, based on the 30-year normal of the 800 m resolution PRISM dataset. Its population density averages 15.3 people per km^2^.

**Fig 8:**
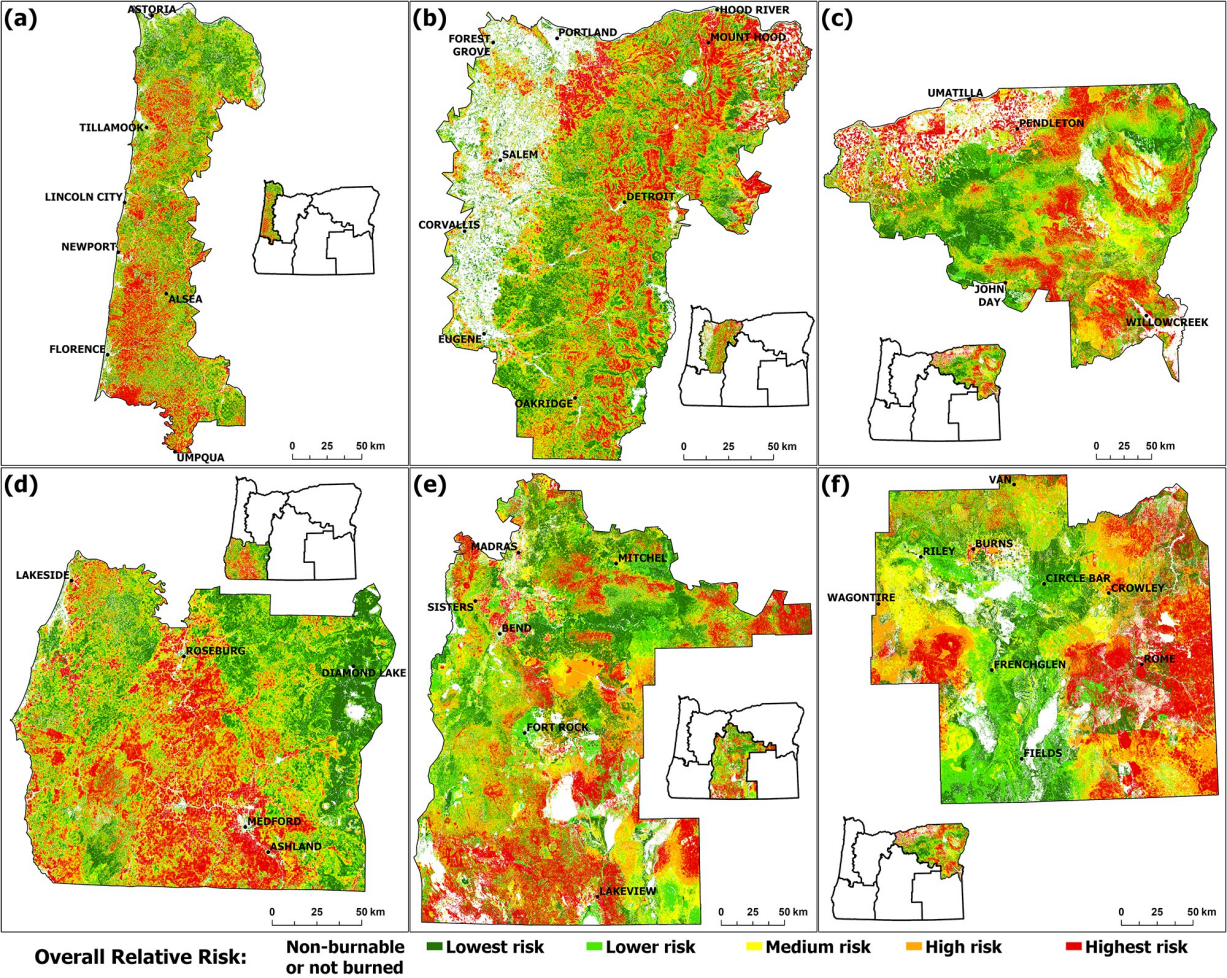
Spatial distribution of overall relative risks in FSA1 to FSA 6 (panel a to f). Transparent areas show grid cells that were classified as non-burnable or not burned by the fire model and consequently have no risk values assigned.

Burn probability and conditional flame length were modeled as low to moderate within FSA 1 (Figs 3a and 4a), and the area had a relatively low IARF (Fig 5a). The ORR ratings specific for FSA 1 (Fig 8a) revealed areas where HVRAs were prevalent, indicating areas of local priority for wildfire risk reduction.

A large portion of the highest potential risk / highest value loss areas within FSA 1 appeared within the Coast Range Mountains. This can be attributed to high fuel loads combined with high timber value, potential habitat loss, and potential loss of threatened, endangered and at-risk species.

**Fig 9:**
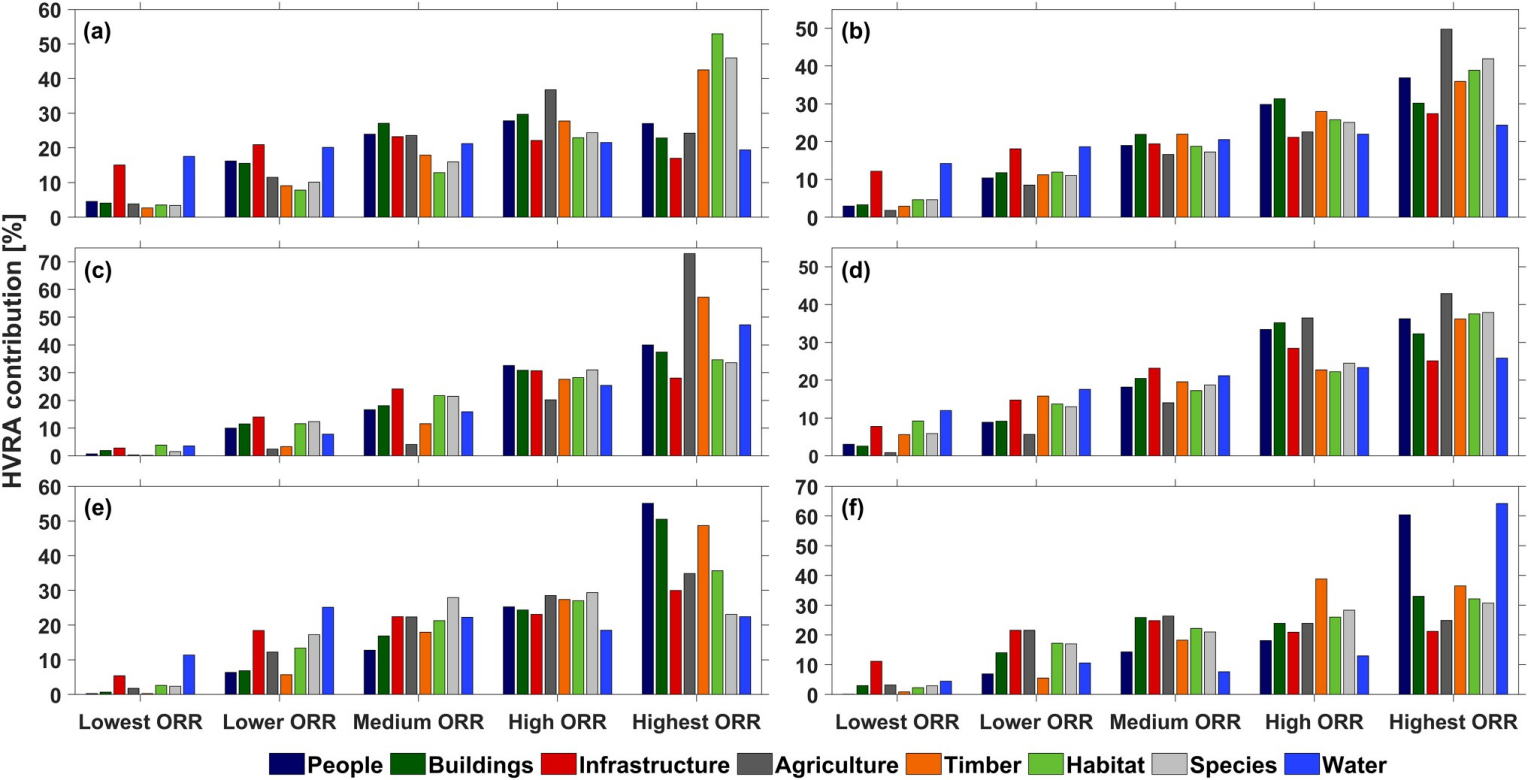
Percent ORR distribution across all risk classes with respect to the HVRA main categories for FSA 1 to 6 (panel a to f).

Fig 9 shows the contributions of each ORR class to the total value loss of each main HVRA group. The Infrastructure and Water HVRA main categories both contributed ca. 20% of the value loss across all risk classes, indicating their homogeneous distribution across different fire regimes in FSA 1. The Timber, Habitat, and Species HVRAs were the primary contributors to highest risk ratings within this FSA.

#### 3.2.2 FSA 2: Willamette Valley and Cascades

FSA 2 covers 32628 km^2^. Its strong west-east gradients in climate and topography resulted in the largest variety of climate and vegetation of all FSAs. The western part of FSA 2 includes the Willamette Valley, where over 70% of the state’s population resides. Agriculture, forested areas, and urban areas dominate the land cover and use. The eastern portion of FSA 2 includes the northern segment of the Oregon Cascade Mountain Range. This densely forested area’s western slopes are dominated by Douglas-fir (*Pseudotsuga menziesii*). Ponderosa pine (*Pinus ponderosa*) dominates its drier eastern slopes, which lie within the range’s rain shadow [73, 74]. FSA 2’s average annual air temperature is 9.4°C and its 30-year mean annual precipitation is 1552 mm. Its population density averages 87.1 people per km^2^, by far the highest of all FSAs.

The west-east gradient is noticeable for the burn probably of this FSA (Fig 3b). Its most western extent includes significant areas modeled as having a burn probability of 0, as these grid cells were categorized as non-burnable or not burned areas such as non-vegetated surfaces, water bodies, irrigated agricultural fields, or built-up areas for which no fuel model existed. Burn probability, in general, increased as in the more easterly extents of the FSA. The IARF peaked in the norther portion of FSA 2 (Fig 5b), on the eastern slopes of the Cascades and around Mount Hood—primarily attributable to insect and disease issues.

The midsection of the FSA was rated at elevated ORR due in large part due to predominance of agricultural HVRAs, while its dense population led to high ORR ratings associated with people-related HVRAs near cities, towns, WUI zones, and related infrastructure. Cities had a particularly striking effect on the FSA’s modeled ORR (Fig 8b). For instance, a large (1020 km^2^) region southeast of Portland ranked in the highest ORR category due in part to its high burn probability and high conditional flame length (related to timber conditions) and amplified by HVRA-specific valuations associated with towns and infrastructure. Similarly, areas near the outskirts of Eugene and Salem rated within the highest ORR class because of the susceptibility to wildfire of HVRAs in those suburban and WUI zones. Zones of highest risk extend east of these cities, where highways and associated WUI zones lead into forested areas within the western Cascades foothills. The highest risk locations correspond well with recorded losses of houses and infrastructure during Oregon’s 2020 fire season, which devastated rural communities along those highways [10].

#### 3.2.3 FSA 3: Northeast

FSA 3 covers an area of 55641 km^2^ and includes the Columbia Basin, with its vast grasslands and agriculture areas, as well as the Blue and Wallowa Mountains. Its population density is low (only 3.1 people per km^2^). Annual average precipitation is relatively low (517 mm) and air temperature averages 8.1°C. Although fuel reduction treatments have been conducted for decades in this region’s forested areas, they remain prone to wildfires due to recurring summer droughts and frequent lightning storms [75–77].

Burn probability was relatively high to very high in much of FSA 3 (Fig 3c). This was particularly evident in the Columbia Basin region (northern extent of the FSA), which is subject to dry and warm summer conditions in combination with high wind speeds of the Columbia Gorge gap flow [78]. Very high burn probability was also evident in the FSA’s high desert southeastern extent. High burn probability was also predicted for much of the FSA’s forested mountain areas, which also featured the region’s highest anticipated flame lengths (Figs 3c and 4c). Additional risk factors (in this case long-term drought and forest health) were evident throughout this (Fig 5c).

The resulting ORR ratings (Fig 8c) were heavily influenced by the underlying burn probabilities, conditional flame lengths, and IARF. The HVRAs, particularly those for agriculture, timber, and habitat, amplified the ratings (Fig 9c).

#### 3.2.4 FSA 4: Southwest

FSA 4 covers a total area of 33780 km^2^, of which 67% is covered by forest. In addition to timber management, significant land uses include agriculture and recreation (e.g., Crater Lake, Oregon’s only National Park). The mean annual air temperature is 10.3°C and annual precipitation averages 1489 mm. The average population density is 14.8 people per km^2^, with most of the population residing in smaller towns along the coast and in the few urban centers of Roseburg, Medford, Grants Pass, and Ashland that are scattered across the otherwise sparsely populated region.

Due to recurring summer droughts, the densely forested Klamath and Siskiyou Mountains are particularly prone to wildfire. Furthermore, the topography facilitates high wind speeds through funnel and slope effects that intensify the heat, expanding the spread and growth of wildfires [79, 80]. Accordingly, FSA 4 has experienced some of the largest and most damaging wildfires on record in Oregon, including the Biscuit Fire in 2002 [81]. It was significantly affected during the 2020 wildfire season [10].

Burn probabilities were modeled as high for much of the FSA (very high near Ashland), and it had the greatest concentration of very high conditional flame lengths of all the FSAs (Figs 3d and 4d). The portion of the IARF relating to drought and forest health were high within the FSA’s eastern extent, and pockets of high IARF values appeared along its western extent associated with Gorse invasion (Fig 5d).

Large, continuous areas of highest ORR are noticeable in the southern central section of FSA 4 (Fig 8d). The Timber, Habitat and Species HVRA main groups contributed significantly to the ORR values across all ORR classes of FSA 4, whereas risks and associated value losses that affect the Agriculture, People, and Buildings main groups were key factors in the high and highest ORR grid cells (Fig 9d). Agriculture, concentrated around the cities of Medford and Roseburg, also contributed to heightened ORR, as did their associated Building and People HVRAs. This means those HVRAs are mostly located within areas of high burn probabilities and conditional flame length, observations that should be of concern to local emergency managers.

#### 3.2.5 FSA 5: Central

With an area of 63023 km^2^ the central FSA is the largest region and incorporates a variety of land cover types associated with its lengthy north-south extent. The western portions of FSA 5 are characterized by vegetation and climate representative of the eastern slopes of the Cascade Mountain Range. The vegetation cover in this forested area is dominated by subalpine species at higher elevations, transitioning through mixed conifer forests to lodgepole and ponderosa pine at lower elevations. Eastward, the vegetation is dominated by open stands of western juniper (*Juniperus occidentalis*). Shrublands of big sagebrush (*Artemisia tridentata)* and bitterbrush (*Purshia tridentata*) dominate as the climate gets drier toward the east.

The eastern extent of FSA 5 exhibits dry and hot summers with most precipitation observed from November through April [82]. The annual precipitation averages 460 mm and the 30-year mean air temperature is 7.2°C. With 4.8 people per km^2^ the area is sparsely populated.

The city of Bend is the FSA’s only large urban center. Its population of roughly 98,000 residents, significant infrastructure, and industry make Bend the socio-economical supply center of the otherwise rural region. Bend exhibits a growing wildland urban interface which makes its outskirts particularly susceptible to wildfire threats [4, 83].

Burn probabilities were modeled as high to very high across the FSA except for three areas associated with higher elevation forests (Fig 3e). Conditional flame lengths were typically low to moderate, with exceptions in the FSA’s southern and northern extents (Fig 4e). The IARF, however, was elevated throughout much of the FSA, reflecting long-term drought and forest health conditions (Fig 5e).

Fig 8e shows highest ORR to the WUI areas north, east, and south of Bend, driven by People and Buildings HVRAs, and numerous areas of high or highest risk associated with Timbered Areas (Fig 9e) in the Eastern Cascades Slopes and Foothill ecoregion [84, 85].

An extensive area (ca. 11000 km^2^) around and east of Klamath Falls, characterized by timber (70%) and agricultural land use (30%), was rated primarily within the highest ORR class (Fig 8e). Between and adjacent to forested areas, bitterbrush dominates in the western half while juniper and sagebrush dominates in the eastern portion of this area.

#### 3.2.6 Southeast FSA 6

FSA 6 covers an area of 45890 km^2^ and incorporates the Oregon High Desert region located in the northwest of the Great Basin. This semiarid area is characterized by volcanic plains broken by local mountain ranges typical of the Great Basin. The open steppe is dominated by bitterbrush (*Purshia tridentata*) and sagebrush (*Artemisia tridentata*), with interspersed rabbit brush (*Chrysothamnus spp*.) and bunchgrasses *(Festuca*, *Poa*, *Eriocoma*, and *Elymus spp*.). The northwestern corner of FSA 6 includes ca. 2000 km^2^ of the southern Blue Mountains ecoregion, where the land cover changes to western juniper and ponderosa pine woodlands. Average annual precipitation within the FSA is 321 mm and the air temperature averages 8.0°C. The area is the least populated FSA in Oregon, with only 0.3 people per km^2^.

The burn probability values show large areas with highest values in the FSA’s eastern extents (Fig 3f). Rangeland is the primary agricultural land use within this segment of FSA 6. The relative low abundance of timber resulted in low overall conditional flame lengths. The area has been affected by large grassland fires attributed to its semiarid climate and an increased spread of invasive species [86]. IARF values associated with long-term drought were of significance in portions of the FSA, and the forested area in the FSA’s northwestern extent was subject to insects and disease (Fig 5f).

Drinking Water from Groundwater Sources is highly valuable in this predominantly semi-arid FSA and exposed to the highest ORR to an extent that exceeds the associated value loss of all other FSAs due to the relative shortage and importance of ground water in this region (Fig 9f). The FSA’s main population centers of Burns and Hines, co-located in the northwestern quadrant of FSA 6, have combined populations of about 4300 inhabitants. Although small, their associated HVRAs shifted the ORR in their vicinities to the highest risk category. Some unnaturally circular-shaped zones with the highest ORR classification in FSA 6 (Fig 8f) are caused by habitat areas allocated for endangered, threatened, and at-risk species found in those areas.

### 3.3 Landscape-scale validation of risk analyses

**Fig 10:**
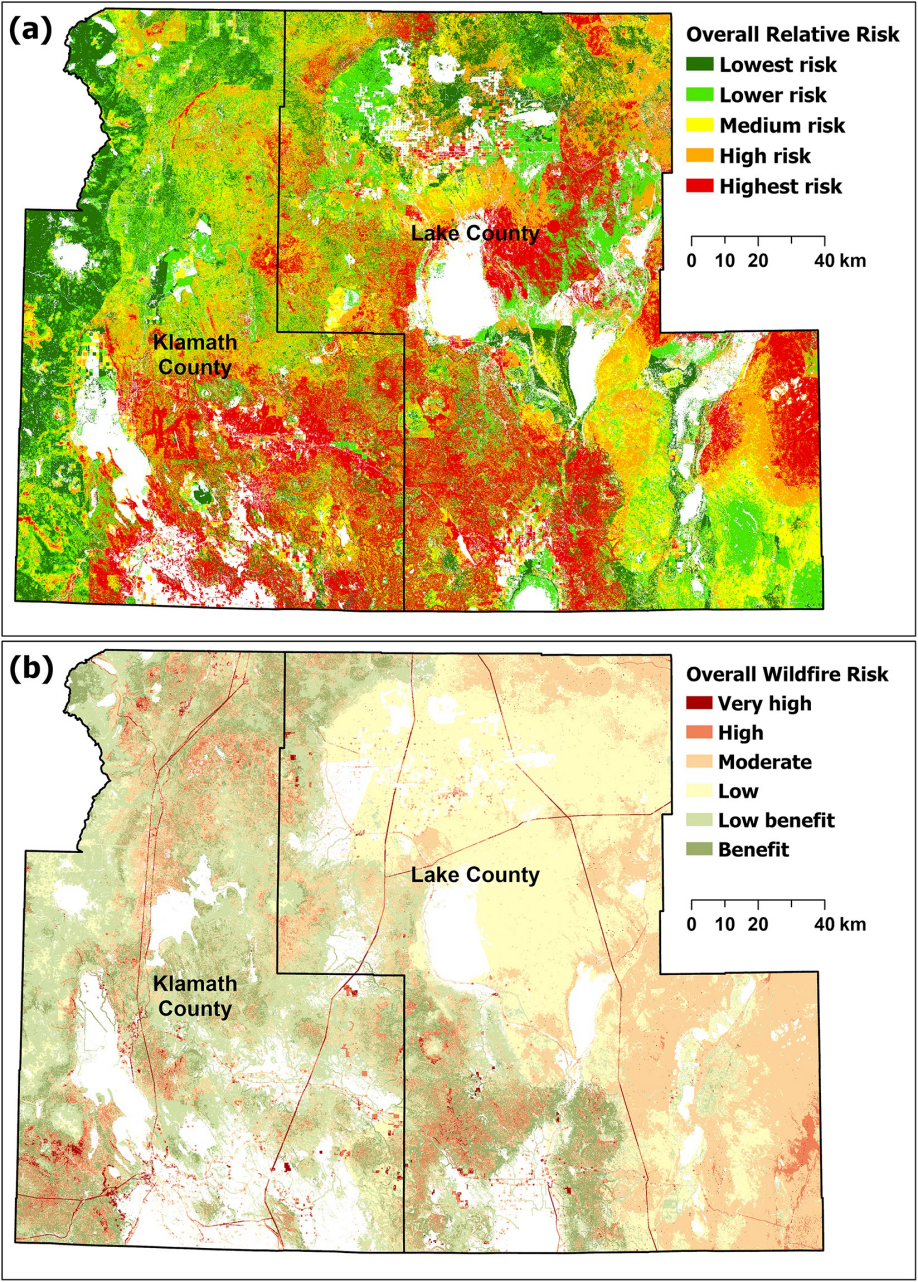
Regional comparison for Klamath and Lake County Oregon between overall relative risk as assigned by EFPRRA (a) and the overall wildfire risk calculated by QWRA 2018 [16] (b).

Klamath and Lake Counties, Oregon, cover an area of over 3.64 million hectares, of which over 70% is federally managed and the remainder primarily privately managed. The EFPRRA projects an ORR in the highest risk category for much of that same area (Fig 10a) whereas the QWRA results indicate a low risk for all of Klamath and Lake County (Fig 10b). QWRA results indicate a low risk for all of Klamath and Lake County (Fig 10b), whereas the EFPRRA projects ORR in the highest risk category for much of that same area (Fig 10a).

From 2015 to 2021, the region completed three landscape-scale, cross-boundary projects designed to implement the goals of the National Cohesive Wildland Fire Management Strategy (https://www.forestsandrangelands.gov/strategy/). Under the auspices of the Klamath Lake Forest Health Partnership (KLFHP) (https://www.klfhp.org/), a non-profit organization with diverse local and regional public/private partners, over 364000 hectares (also approximately 70% public and 30% private) were treated to mitigate fire risk to resources and assets. Private land areas within the projects were extensively mapped during treatment planning using 1-meter resolution imagery [86], and later ground-validated, to identify landcover type, vegetation density, and age of dominant vegetation. Wildfire risk mitigation plans were then developed based on these data.

**Fig 11:**
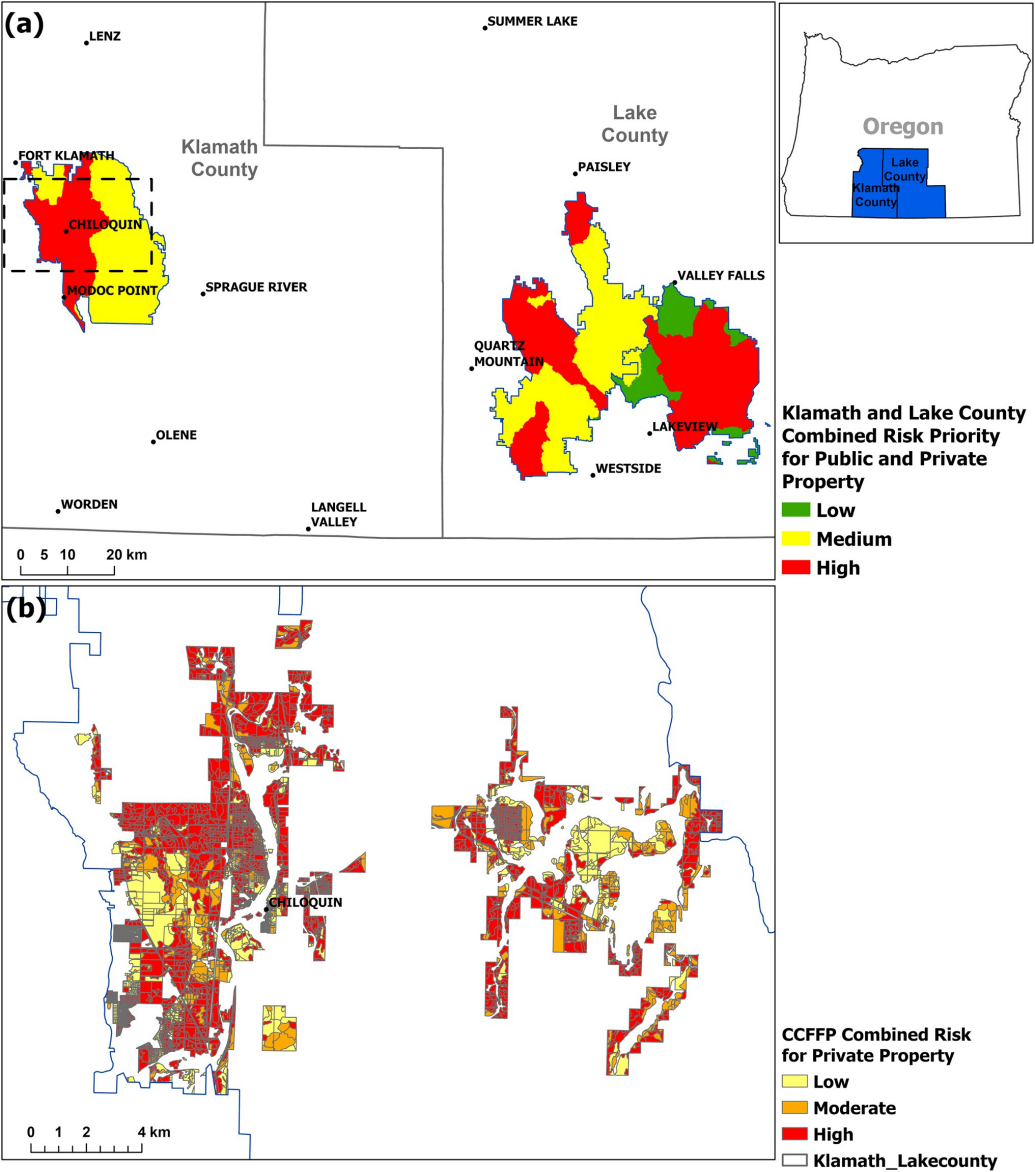
Ground-based fire risk assessed on the landscape scale on private lands within the Chiloquin Community Forest and Fire Project.

Fig 11 illustrates the fire risk assessed on approximately 16000 hectares of private lands within the Chiloquin Community Forest and Fire Project (CCFFP), surrounding the community of Chiloquin, north of Klamath Falls [87]. Note the preponderance of area categorized as high risk, and its alignment with risk indicated by the EFPRRA (Fig 10a). The relative risk assessment completed is consistent with the inventory and mapping conducted during the landscape assessments. This effort is used as a tool for risk assessment and encourage it to be used with up-to-date landform layers and locally derived HVRA metrics.

## 4. Discussion

The western United States benefits from several excellent fire risk models, each with its own emphasis. The QWRA [16, 69] and West Wide Wildfire Risk Assessment [88] focused on statewide or a multi-state evaluation of overall fire risk and implications for core sets of resources and assets. Other models facilitate analysis of tradeoffs among ecosystem services, fire suppression, and/or mitigation of wildfire impacts [89–91] or focus on risk assessments specifically for human populations [17].

The EFPRRA presented here was designed to allow customized risk assessments at spatial scales varying from a subregion of the state (FSA) to individual municipalities, as a tool tailored to the needs of the Extension Service Fire Program. The refined meteorological data available from our analysis of RAWS allows the model to reflect weather patterns most relevant to our objectives. In our case, we chose worst-case scenarios with respect to heat and drought, which align with current climate trends [7, 8, 36] and are likely appropriate for future modeling efforts. While we drew baseline land condition data from the *LandFire* data repository, other local data could be substituted leading to truly customized and current burn probabilities and expected flame lengths. This allows the model to reflect landcover changes associated with wildfires or forest management activities. Our risk model builds in additional capacity through integration of additional risks factors. Its approach is straightforward and can be refined for risks specific to any locality or region. Finally, and perhaps most importantly, our method of valuing resources and assets (HVRAs) is fully intended to be adjusted where needed to best represent stakeholders within their regions of interest.

The HVRAs incorporated into this model broadly represent those impacted by wildfire within Oregon, but they are by no means all-inclusive nor are they exclusive. Users can add or remove HVRAs from the model and can modify weighting factors to better represent constituent perspectives. Capacity for customization was a key consideration in the Extension Fire Program’s decision to develop this risk assessment model.

Individual private landowners, local governments, or nation states are not able to significantly modify underlying climate issues (doing so will require systematic and comprehensive global efforts), but they can take more localized steps to mitigate wildfire intensity and risk to people, resources, and assets. For example, active treatments can be applied to reduce vegetative fuel loads and restore ecosystem composition and heterogeneity. Education and regulatory actions can be utilized to minimize unintended ignitions, building codes/requirements adjusted to control housing density in fire-prone areas, and practices promoted that reduce fire risk to homes and home landscapes. Fire risks and implications of wildfire, however, are not limited to any one landowner or land management entity—fires ignore political and ownership boundaries. A significant proportion of land in Oregon is privately owned, and risk levels associated with private lands varied by FSA in our assessment. The percentage of private land assigned to the highest ORR category ranged from 11.8% (FSA 6) to 51.5% (FSA 3), underlining the importance of collaboration across physical, administrative, and ownership boundaries for management and treatments aimed to protect resources from wildfire damage.

Our modelling approach offers Oregon (and other) communities a highly scalable and customizable approach to identifying areas of wildfire risk, as a means of prioritizing risk mitigation efforts. Regardless of whether those communities choose this model or another, we reemphasize the urgency of taking active measures to reduce wildfire risk as the western United States’ forest conditions and changing climate demand concerted and collaborative attention.

## Supporting information

S1 Text Data used for delineation of Fire Service Areas. The 13 gridded variables used for spatial clustering procedure, Reclassification of directional data with respect to fire promoting/enforcing properties. and Parameters for solar irradiance calculations.
(DOCX)

S2 Text Data sources for “buildings with vulnerable people” HVRA. Medical Facilities, Emergency Shelters, Schools and Nursing homes.
(DOCX)

S3 Text Additional data and valuations of “species” sub-categories. Big Game habitat, Salmon habitat, and Valuation of critical habitat for USFWS listed species in Oregon.
(DOCX)

S4 Text List of acronyms. (DOCX)

S1 Table List of crops and values in “agriculture” HVRA dataset. Cultivated acreage and valuations for 66 crops cultivated in Oregon and the corresponding vulnerability/response function classification and valuation applied.
(DOCX)

S2 Table Valuations for the “habitat” HVRA category. Valuation of the habitat HVRA layer from 0 (no or very low value) to 9 (very high value) of 77 specific habitats in Oregon.
(DOCX)

S3 Table Response function values used for overall relative risk calculation. Response function percentage values for FSA-specific risk assessments.
(DOCX)
