# Correction: A quantitative wildfire risk assessment using a modular approach of geostatistical clustering and regionally distinct valuations of assets—A case study in Oregon

**DOI:** 10.1371/journal.pone.0308311

**Published:** 2024-07-31

**Authors:** Andres Schmidt, Daniel Leavell, John Punches, Marco A. Rocha Ibarra, James S. Kagan, Megan Creutzburg, Myrica McCune, Janine Salwasser, Cara Walter, Carrie Berger

In the abstract section, there are a typographical error in the paragraph. The correct paragraph is: The intensity and scale of wildfires has increased throughout the Pacific Northwest in recent decades, especially within the last decade, destroying vast amounts of valuable resources and assets. This trend is predicted to remain or even magnify due to climate change, growing population, and increased housing density. Furthermore, the associated stress of prolonged droughts and change in land cover/land use puts more population at risk. We present results of a multi-phase Extension Fire Program Initiative combining fire model results based on worst-case meteorological conditions recorded at 50 weather stations across Oregon with spatially distinct valuations of resources and assets based on regional ecological and socio-economic conditions. Our study focuses on six different Fire Service Areas covering the state of Oregon. We used a geostatistical approach to find weather stations that provide worst-case meteorological input data on record for representative sub-domains. The results provide regionally distinct assessments of potential value loss by wildfire and show that, depending on the region, 12% to 52% of the highest relative risk areas are on private land. This underscores the need to unite strategies and efforts on the landscape scale by including different landowners, managers, and stakeholders of public land and private land to efficiently address wildfire damage protection and mitigation. Our risk assessments closely agreed with risks identified during landscape-scale ground projects.

The Data Availability statement for this paper is incorrect. The correct statement is: All relevant data are within the paper and its Supporting information files. Additional maps and input data used will be made available at: https://extension.oregonstate.edu/fire-program.

In Fig 1 and Fig 8, the small overview graphic in the lower left of panel (f) is incorrect. Please see the correct Fig 1 and Fig 8 here.

**Fig 1:**
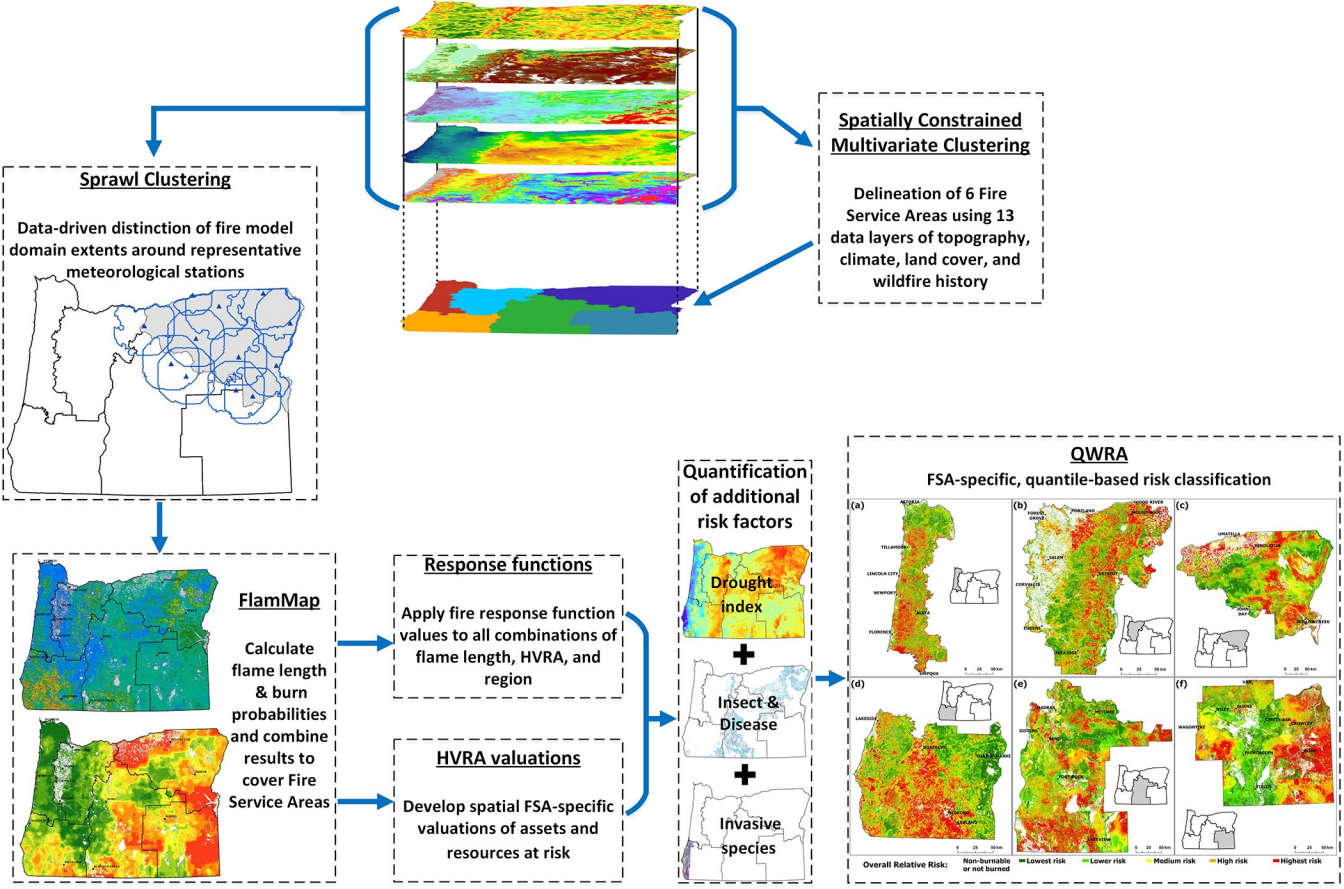
Schematic overview of the processes conducted for the risk assessment.

**Fig 8:**
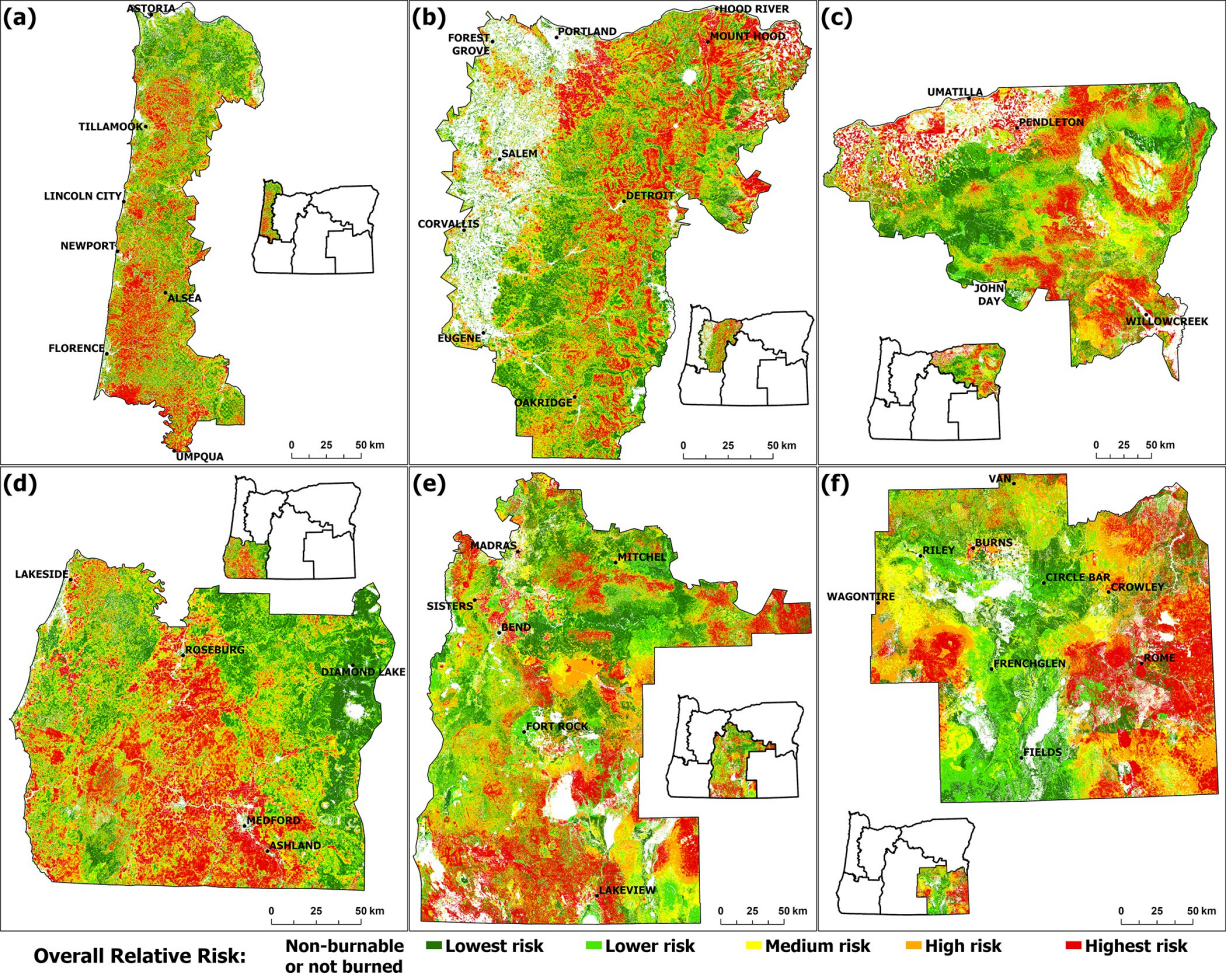
Spatial distribution of overall relative risks in FSA1 to FSA 6 (panel a to f).

Transparent areas show grid cells that were classified as non-burnable or not burned by the fire model and consequently have no risk values assigned.
