# Supplementary material for: A quantitative wildfire risk assessment using a modular approach of geostatistical clustering and regionally distinct valuations of assets—A case study in Oregon
Source: PLoS One. 2022 Mar 8;17(3):e0264826. doi: 10.1371/journal.pone.0264826 (PMC8903305; doi:10.1371/journal.pone.0264826)
Supplement: S1 Text — The 13 gridded variables used for spatial clustering procedure, Reclassification of directional data with respect to fire promoting/enforcing properties. and Parameters for solar irradiance calculations. (DOCX) [file pone.0264826.s001.docx]

**S1 Text Data used for delineation of Fire Service Areas.**

Following redundancy checks based on cross correlations, the final dataset used for the clustering procedure was composed of climate variables, topographic information, land cover data, and historic fire frequency (Table A).

Table A: The 13 gridded variables used for spatial clustering procedure.

| **Variable** | **Data source** |
| --- | --- |
| Minimum air temperature | PRISM (Parameter-elevation Relationships on Independent Slopes Model) 30-year normal values  [http://www.prism.oregonstate.edu/normals (accessed Oct](http://www.prism.oregonstate.edu/normals%20(accessed%20Oct). 1 2020) |
| Maximum air temperature |  |
| Average air temperature |  |
| Minimum vapor pressure deficit |  |
| Maximum vapor pressure deficit |  |
| Annual Precipitation |  |
| Average wind speed (2007-2013)  in 40 m above ground | Wind Prospector, National Renewable Energy Laboratory https://maps.nrel.gov/wind-prospector |
| Historic fire frequency: 1984-2017 | MTBS National Burned Areas Boundaries Dataset 1984 - 2017 <https://www.mtbs.gov/>  [(accessed Oct](http://www.prism.oregonstate.edu/normals%20(accessed%20Oct). 4 2020) |
| Elevation  Aspect | NASA Shuttle Radar Topography Mission (2013), SRTM 30m, Distributed by OpenTopography.  https://doi.org/10.5069/G9445JDF (accessed: Oct. 8 2020) |
| Slope |  |
| Landcover type | NLCD 2016, Multi-Resolution Land Characteristics Consortium  <https://www.mrlc.gov> (accessed: Oct. 8, 2020) |
| Solar radiation: 14-day northern hemisphere mid-latitude maximum (June 21- July 4) | Produced using 30 m SRTM elevation data in combination with ESRI ArcGIS Pro Area Solar Radiation tool |

The original 360-degree classification for orographic aspect data in a 900 m x 900 m horizontal resolution for the State of Oregon was transferred into a classification system consisting of eight main wind direction classes with regard to their specific influences related to wildfire behavior. The first cluster attribute was assigned to the three main classes “Low Reinforcement”, “Medium Reinforcement”, and “High Reinforcement”. To assign numerical values that distinguish the wind sectors, the three main classes were subdivided into levels ranging from 1 to 3, with 1 being the lowest reinforcement category in the respective main class and 3 being the highest.

Table B: Reclassification of directional data with respect to fire promoting/enforcing properties.

| **Original class attributes in 360^◦^ resolution [deg]** | **Sector width [deg]** | **Cardinal Wind direction** | **New classification pertaining to influence on fire conditions** | **Assigned numeric value** |
| --- | --- | --- | --- | --- |
| 337-360,0-21 | 44 | North | Low Reinforcement – Level 2 | 2 |
| 22-66 | 44 | Northeast | Low Reinforcement – Level 3 | 3 |
| 67-111 | 44 | East | Medium Reinforcement – Level 2 | 51 |
| 112-156 | 44 | Southeast | High Reinforcement – Level 1 | 100 |
| 157-201 | 44 | South | High Reinforcement – Level 2 | 101 |
| 202-246 | 44 | Southwest | High Reinforcement – Level 3 | 102 |
| 247-291 | 44 | West | Medium Reinforcement – Level 1 | 50 |
| 292-336 | 44 | Northwest | Low Reinforcement – Level 1 | 1 |

USGS NED ⅓ arc second rasters were downloaded in 1**^◦^** x 1**^◦^** tiles to cover the entire state (42 to 47°N, 117 to 125 °W), and aggregated to 900 x 900 m resolution raster. The projected raster was split into quarter latitude strips and each strip was input into the ArcGIS Pro Area Solar Radiation tool using the average latitude for each strip (Tab C).

Table C: Parameters for solar irradiance calculations.

| **Parameter** | **Selection** |
| --- | --- |
| Time configuration | Multiple days |
| Year | 2019 |
| Start day | 60 (March 1) |
| End day | 273 (September 30) |
| Day interval | 14 |
| Hour interval | 0.5 |
| Topographic parameters:   - Z factor - Slope and aspect input type - Calculation directions | - 1 - From the input surface raster - 32 |
| Radiation parameters   - Zenith divisions - Azimuth divisions - Diffuse model type - Diffuse proportion - Transmissivity | - 8 - 8 - Uniform overcast sky - 0.3 - 0.5 |

For the 30m x 30m solar irradiation layer, the total maximum solar radiation for the state for the 2-week period from June 21 through July 4 with was used.
