# Supplementary material for: A quantitative wildfire risk assessment using a modular approach of geostatistical clustering and regionally distinct valuations of assets—A case study in Oregon
Source: PLoS One. 2022 Mar 8;17(3):e0264826. doi: 10.1371/journal.pone.0264826 (PMC8903305; doi:10.1371/journal.pone.0264826)
Supplement: S2 Text — Medical Facilities, Emergency Shelters, Schools and Nursing homes. (DOCX) [file pone.0264826.s002.docx]

**S2 Text Data sources for “buildings with vulnerable people” HVRA**

Medical Facilities: Medical facilities include Hospitals, Hospital Satellites, Other Medical Facilities and Urgent Care Centers.

Hospitals (2019) from HIFLD: https://hifld-geoplatform.opendata.arcgis.com/.

Hospitals_OHA layer (2014) from Oregon Incident Response Information System (OR-IRIS): https://spatialdata.oregonexplorer.info/geoportal/details;id=165d6af382ec435fb3faa3ca6e0c2736

Hospital_Satellites_OHA layer (2014) from OR-IRIS (OR-IRIS data link above).

Licensed_Medical_Facilities_OHA layer (2015) from OR-IRIS (OR-IRIS data link above).

Urgent_Care_Centers layer (2016) from OR-IRIS (OR-IRIS data link above).

Emergency Shelters:

National_Shelter_System_Facilities layer (2019) from HIFLD: https://hifld-geoplatform.opendata.arcgis.com/

Emergency_Shelters (2015) from OR-IRIS: https://spatialdata.oregonexplorer.info/geoportal/ details;id=165d6af382ec435fb3faa3ca6e0c2736.

Schools:

EDUCATIONAL_LOCATIONS (2016) from Oregon Dept. of Human Services & Oregon Health Authority: https://spatialdata.oregonexplorer.info/geoportal/ details;id=1270fe6e833f4d0eabacc71300069738.

Nursing Homes:

Nursing_Homes layer (2019) from HIFLD: https://hifld-geoplatform.opendata.arcgis.com/.
