# Supplementary material for: A quantitative wildfire risk assessment using a modular approach of geostatistical clustering and regionally distinct valuations of assets—A case study in Oregon
Source: PLoS One. 2022 Mar 8;17(3):e0264826. doi: 10.1371/journal.pone.0264826 (PMC8903305; doi:10.1371/journal.pone.0264826)
Supplement: S3 Text — Big Game habitat, Salmon habitat, and Valuation of critical habitat for USFWS listed species in Oregon. (DOCX) [file pone.0264826.s003.docx]

**S3 Text Additional data and valuations of “species” sub-categories**

Big Game habitat summarizes maps of winter range habitat for deer and for elk in Oregon from the Oregon Department of Fish and Wildlife (https://nrimp.dfw.state.or.us/ DataClearinghouse/default.aspx?p=202&XMLname=885.xml). The raster contains values from 0 to 2, with 0 values where no deer or elk winter range habitat is mapped, a value of 1 where there is winter habitat for either deer or elk, and a value of 2 for areas that provide both deer and elk winter habitat. This dataset has a maximum value of 2 but is scaled from 0-9, recognizing that other species in this VAR category are more valuable and vulnerable to wildfire.

Salmon habitat identifies essential habitat for salmonids from a 2015 layer provided by Department of State Lands (DSL) and Oregon Department of Fish and Wildlife. This dataset is available online as an ArcGIS service (https://chetco-new.dsl.state.or.us/arcgis/rest/ services/Maps/ESH_State/MapServer) but was obtained directly from DSL for this project. This map was used in lieu of the National Oceanic and Atmospheric Administration (NOAA) critical habitat maps of federally listed salmon primarily because the ORBIC EOR database (described under Threatened, Endangered and At-Risk Species) includes salmon streams and rivers in Oregon, and significantly overlaps with these NOAA maps. Additionally, the Essential Salmon Habitat map is specifically defined by Oregon law as representing the significant salmon areas in the State.

Table A: Valuation of critical habitat for USFWS listed species in Oregon.

| **Common Name** | **Scientific Name** | **Status** | **Value** |
| --- | --- | --- | --- |
| Marbled murrelet | *Brachyramphus marmoratus* | Threatened | 2 |
| Bull Trout | *Salvelinus confluentus* | Threatened | 0 |
| Oregon spotted frog | *Rana pretiosa* | Threatened | 1 |
| Willamette daisy | *Erigeron decumbens* | Endangered | 2 |
| Fender's blue butterfly | *Icaricia icarioides fenderi* | Endangered | 2 |
| Malheur wire-lettuce | *Stephanomeria malheurensis* | Endangered | 2 |
| Kincaid's Lupine | *Lupinus sulphureus* ssp. *kincaidii* | Threatened | 2 |
| Borax Lake chub | *Gila boraxobius* | Endangered | 0 |
| Northern spotted owl | *Strix occidentalis caurina* | Threatened | 2 |
| Oregon silverspot butterfly | *Speyeria zerene hippolyta* | Threatened | 2 |
| Vernal pool fairy shrimp | *Branchinecta lynchi* | Threatened | 2 |
| Shortnose Sucker | *Chasmistes brevirostris* | Endangered | 0 |
| Warner sucker | *Catostomus warnerensis* | Threatened | 0 |
| Cook's lomatium | *Lomatium cookii* | Endangered | 2 |
| Western snowy plover | *Charadrius nivosus nivosus* | Threatened | 1 |
| Large-flowered woolly meadowfoam | *Limnanthes pumila* ssp. *grandiflora* | Endangered | 2 |
| Streaked Horned lark | *Eremophila alpestris strigata* | Threatened | 2 |
| Taylor's (Whulge) Checkerspot | *Euphydryas editha taylori* | Endangered | 2 |
