# Supplementary material for: A quantitative wildfire risk assessment using a modular approach of geostatistical clustering and regionally distinct valuations of assets—A case study in Oregon
Source: PLoS One. 2022 Mar 8;17(3):e0264826. doi: 10.1371/journal.pone.0264826 (PMC8903305; doi:10.1371/journal.pone.0264826)
Supplement: S4 Text — (DOCX) [file pone.0264826.s004.docx]

**S4 Text List of Acronyms**

**BLM** Bureau of Land Management

**CCFFP** Chiloquin Community Forest and Fire Project

**DSL** Department of State Lands

**EFPRRA** Extension Fire Program Relative Risk Assessment

**EOR**  Element Occurrence Record

**FSA** Fire Service Area

**GAP** Gap Analysis Program

**GNN**  Gradient Nearest Neighbor

**HIFLD** Department of Homeland Security through the Homeland Infrastructure Foundation-
Level Data database

**HVRA**  Highly Valued Resources and Assets

**IARF** Integrated Additional Risks Factor

**MTBS** Monitoring Trends in Burn Severity

**OAR**  Oregon Administrative Rules

**ODA**  Oregon Department of Agriculture

**ODFW**  Oregon Department of Fisheries and Wildlife

**ORBIC**  Oregon Biodiversity Information Center

**ORR**  Overall Relative Risk

**OSU**  Oregon State University

**PAD**  Protected Areas Database

**PRISM** Parameter-elevation Regressions on Independent Slopes Model

**QWRA**  Quantitative Wildfire Risk Assessment

**RAWS**  Remote Automatic Weather Stations

**SCMC**  Spatially Constrained Multivariate Clustering

**SCPDSI** Self-Calibrated Palmer Drought Severity Index

**USDA** United States Department of Agriculture

**USFS**  United States Forest Service

**WUI** Wildland-Urban Interface
