# Supplementary material for: A quantitative wildfire risk assessment using a modular approach of geostatistical clustering and regionally distinct valuations of assets—A case study in Oregon
Source: PLoS One. 2022 Mar 8;17(3):e0264826. doi: 10.1371/journal.pone.0264826 (PMC8903305; doi:10.1371/journal.pone.0264826)
Supplement: S1 Table — Cultivated acreage and valuations for 66 crops cultivated in Oregon and the corresponding vulnerability/response function classification and valuation applied. (DOCX) [file pone.0264826.s005.docx]

**S1 Table** **List of crops and values in “agriculture” HVRA dataset**

Table S3: Cultivated acreage and valuations for 66 crops cultivated in Oregon and the corresponding vulnerability/response function classification and valuation applied.

| **Crop** | **Area cultivated in Oregon (km^2^)** | **Value class** | **Response function class (0-3)** |
| --- | --- | --- | --- |
| Other Tree Crops | 429.40 | 9 | 3 |
| Christmas Trees | 33.56 | 9 | 3 |
| Winter Wheat | 3019.86 | 6 | 3 |
| Barley | 149.81 | 6 | 3 |
| Oats | 54.40 | 6 | 3 |
| Hops | 49.20 | 6 | 2 |
| Grapes | 42.30 | 6 | 2 |
| Caneberries | 0 | 6 | 2 |
| Alfalfa | 2482.51 | 4 | 2 |
| Corn | 340.28 | 3 | 1 |
| Potatoes | 170.68 | 3 | 1 |
| Onions | 98.11 | 3 | 1 |
| Pears | 97.36 | 3 | 1 |
| Cherries | 91.40 | 3 | 1 |
| Peas | 81.46 | 3 | 1 |
| Mint | 75.89 | 3 | 1 |
| Sweet Corn | 75.61 | 3 | 1 |
| Blueberries | 43.71 | 3 | 1 |
| Sugar beets | 40.99 | 3 | 1 |
| Apples | 16.39 | 3 | 1 |
| Strawberries | 1.75 | 3 | 1 |
| Other Hay/ Non-Alfalfa | 1441.82 | 2 | 2 |
| Spring Wheat | 246.66 | 2 | 1 |
| Dry Beans | 82.36 | 2 | 2 |
| Triticale | 76.49 | 2 | 2 |
| Rye | 11.85 | 2 | 2 |
| Canola | 9.66 | 2 | 2 |
| Chick Peas | 8.44 | 2 | 2 |
| Sunflower | 2.54 | 2 | 2 |
| Millet | 0.99 | 2 | 2 |
| Dbl. Crop Winter wheat / Corn | 0.86 | 2 | 1 |
| Sod/Grass Seed | 2140.99 | 1 | 1 |
| Clover/Wildflowers | 170.13 | 1 | 1 |
| Dbl. Crop Triticale/Corn | 21.63 | 1 | 1 |
| Mustard | 4.10 | 1 | 1 |
| Plums | 2.55 | 1 | 1 |
| Vetch | 1.29 | 1 | 1 |
| Durum Wheat | 0.87 | 1 | 1 |
| Peaches | 0.29 | 1 | 1 |
| Pop. or Orn. Corn | 0.02 | 1 | 1 |
| Tomatoes | 0 | 1 | 1 |
| Fallow/Idle Cropland | 2863.22 | 0 | 0 |
| Other Crops | 44.23 | 0 | 0 |
| Carrots | 21.46 | 0 | 0 |
| Radishes | 18.72 | 0 | 0 |
| Squash | 6.80 | 0 | 0 |
| Garlic | 5.12 | 0 | 0 |
| Turnips | 4.83 | 0 | 0 |
| Watermelons | 3.55 | 0 | 0 |
| Greens | 2.39 | 0 | 0 |
| Herbs | 2.04 | 0 | 0 |
| Flaxseed | 1.65 | 0 | 0 |
| Soybeans | 1.53 | 0 | 0 |
| Sorghum | 1.51 | 0 | 0 |
| Cauliflower | 1.42 | 0 | 0 |
| Broccoli | 1.40 | 0 | 0 |
| Gourds | 0.99 | 0 | 0 |
| Sweet Potatoes | 0.89 | 0 | 0 |
| Peppers | 0.67 | 0 | 0 |
| Misc. Vegetables & Fruits | 0.50 | 0 | 0 |
| Pumpkins | 0.23 | 0 | 0 |
| Lettuce | 0.22 | 0 | 0 |
| Speltz | 0.19 | 0 | 0 |
| Cabbage | 0.06 | 0 | 0 |
| Dbl. Crop Winter wheat/Sorghum | 0.05 | 0 | 0 |
| Cantaloupes | 0.02 | 0 | 0 |
