# Supplementary material for: A quantitative wildfire risk assessment using a modular approach of geostatistical clustering and regionally distinct valuations of assets—A case study in Oregon
Source: PLoS One. 2022 Mar 8;17(3):e0264826. doi: 10.1371/journal.pone.0264826 (PMC8903305; doi:10.1371/journal.pone.0264826)
Supplement: S2 Table — Valuation of the habitat HVRA layer from 0 (no or very low value) to 9 (very high value) of 77 specific habitats in Oregon. (DOCX) [file pone.0264826.s006.docx]

**S2 Valuations for the “habitat” HVRA category**

Table S4: Valuation of the habitat HVRA layer from 0 (no or very low value) to 9 (very high value) of 77 specific habitats in Oregon.

| **Habitat** | **Area coverage in Oregon (km^2^)** | **Value class** | **Justification** |
| --- | --- | --- | --- |
| Douglas-fir - Western Hemlock old-growth | 6096.23 | 9 | old-growth forest, vulnerable and very valuable |
| Mixed Conifer (White, Douglas-fir-Pine) old-growth | 2651.47 | 9 | old-growth forest, vulnerable and very valuable |
| Silver Fir - Mountain Hemlock old-growth | 1784.54 | 9 | old-growth forest, vulnerable and very valuable |
| Siskiyou Mixed Conifer old-growth | 1136.21 | 9 | old-growth forest, vulnerable and very valuable |
| Mixed Hardwood - Conifer old-growth | 556.75 | 9 | old-growth forest, vulnerable and very valuable |
| Ponderosa Pine old-growth | 212.16 | 9 | old-growth forest, vulnerable and very valuable |
| Mixed Oak - Conifer old-growth | 193.73 | 9 | old-growth forest, vulnerable and very valuable |
| Coastal Spruce, Cedar or Redwood old-growth | 95.09 | 9 | old-growth forest, vulnerable and very valuable |
| Spruce - Subalpine Fir old-growth | 81.22 | 9 | old-growth forest, vulnerable and very valuable |
| Mixed Conifer (White or Douglas-fir/Pine) mature | 8395.67 | 7 | mature forest, vulnerable and valuable |
| Ponderosa Pine mature | 7620.63 | 7 | includes open & closed - vulnerable forests |
| Douglas-fir - Western Hemlock mature | 3059.37 | 7 | mature forest, vulnerable and valuable |
| Silver Fir - Mountain Hemlock mature | 2055.33 | 7 | mature forest, vulnerable and valuable |
| Siskiyou Mixed Conifer mature | 1487.37 | 7 | mature forest, vulnerable and valuable |
| Mixed Hardwood - Conifer mature | 657.66 | 7 | mature forest, vulnerable and valuable |
| Mixed Oak - Conifer mature | 217.65 | 7 | mature forest, vulnerable and valuable |
| Coastal Spruce, Cedar or Redwood mature | 47.35 | 7 | mature forest, vulnerable and valuable |
| Big Sagebrush fair - good | 31304.08 | 6 | very sensitive and valuable steppe |
| Lodgepole Pine mature | 2349.09 | 6 | very sensitive forest over 30 years old |
| Western Juniper old-growth | 2150.36 | 6 | older woodland sensitive to fire, limited area |
| Low Sagebrush fair - good | 10198.57 | 5 | somewhat sensitive and valuable steppe |
| Mountain Big Sagebrush fair - good | 3317.36 | 5 | somewhat sensitive and valuable steppe |
| Spruce - Subalpine Fir medium to mature | 2864.00 | 5 | very sensitive forest 30 to 80 years old |
| Douglas-fir - Western Hemlock medium | 15333.10 | 4 | very sensitive, valuable forest, 30 to 80 years old |
| Alkali and Desert Grasslands | 509.89 | 4 | uncommon and converts to annuals after fire |
| Columbia Basin Grasslands and Prairie | 14557.89 | 3 | often recovers fine, occasionally not |
| Mixed Conifer (White or Douglas-fir/Pine) medium | 13735.96 | 3 | sensitive forest 30 to 80 years old |
| Ponderosa Pine medium | 9005.70 | 3 | sensitive forest between 30 and 80 years old |
| Mixed Hardwood - Conifer medium | 5312.43 | 3 | sensitive forest between 30 and 80 years old |
| Coastal and Valley Riparian | 4044.53 | 3 | somewhat sensitive and slightly vulnerable |
| Siskiyou Mixed Conifer medium | 3139.10 | 3 | sensitive forest between 30 and 80 years old |
| Interior Lowland and Foothill Riparian | 2827.80 | 3 | somewhat sensitive and slightly vulnerable |
| Oak | 2130.32 | 3 | somewhat sensitive and slightly vulnerable |
| Silver Fir - Mountain Hemlock medium | 1388.11 | 3 | sensitive forest between 30 and 80 years old |
| Coastal Spruce, Cedar or Redwood medium | 724.16 | 3 | sensitive forest between 30 and 80 years old |
| Marshes, Bogs and Emergent Wetlands | 3004.49 | 2 | only slightly vulnerable |
| Mixed Oak - Conifer young to medium | 1724.61 | 2 | somewhat sensitive and slightly vulnerable |
| Canyon & Montane Shrubland | 1240.00 | 2 | requires fire but some fire-free intervals |
| Quaking Aspen | 1210.92 | 2 | somewhat sensitive and slightly vulnerable |
| Lowland Woody Wetlands and Swamps | 625.29 | 2 | not very vulnerable but require recovery |
| Chaparral | 430.97 | 2 | requires fire but some fire-free intervals |
| Montane Grasslands and Dry Meadows | 421.43 | 2 | not very vulnerable but require recovery |
| Coastal and Valley Grasslands | 292.36 | 2 | not very vulnerable but require recovery |
| Subalpine Parkland | 123.53 | 2 | not very vulnerable but require recovery |
| Big Sagebrush poor | 9434.51 | 1 | degraded habitat |
| Douglas-fir - Western Hemlock young | 5623.01 | 1 | forests less than 30 years old |
| Low Sagebrush poor | 1270.23 | 1 | degraded habitat |
| Mixed Hardwood - Conifer young | 836.61 | 1 | forests less than 30 years old |
| Ponderosa Pine young | 818.57 | 1 | forests less than 30 years old |
| Lodgepole Pine young | 541.67 | 1 | forests less than 30 years old |
| Mixed Conifer (White or Douglas Fir/Pine) young | 440.75 | 1 | forests less than 30 years old |
| Alpine | 383.12 | 1 | not very vulnerable |
| Siskiyou Mixed Conifer young | 343.25 | 1 | forests less than 30 years old |
| Mountain Big Sagebrush poor | 302.94 | 1 | degraded habitat |
| Montane Wetlands | 214.24 | 1 | not very vulnerable |
| Coastal Spruce, Cedar or Redwood young | 159.92 | 1 | forests less than 30 years old |
| Coastal Dunes and Beaches | 129.43 | 1 | not very vulnerable |
| Inland Dunes | 101.97 | 1 | not very vulnerable |
| Spruce - Subalpine Fir young | 50.58 | 1 | forests less than 30 years old |
| Silver Fir - Mountain Hemlock young | 50.06 | 1 | forests less than 30 years old |
| Young Western Juniper | 13538.89 | 0 | invading juniper, agencies seek to remove |
| Cultivated Crops | 12533.30 | 0 | addressed by “Agriculture” HVRA values |
| Exotic Grasslands and Annuals | 7326.42 | 0 | non-native habitat |
| Early Shrub-Tree | 6531.40 | 0 | young clear cuts |
| Pasture or Hay | 6514.59 | 0 | addressed by agriculture HVRA values |
| Suburban (Moderate Intensity Developed) | 3546.38 | 0 | addressed by “Buildings and People” HVRA values |
| Open Water (Big Rivers and Reservoirs) | 3083.21 | 0 | not vulnerable |
| Salt Desert Scrub | 2742.72 | 0 | not vulnerable |
| Burns | 1651.46 | 0 | not vulnerable |
| Playa and Barren Ash | 1524.88 | 0 | not vulnerable |
| Rural Residential (Low Intensity Developed) | 1522.40 | 0 | addressed by “Buildings and People” HVRA values |
| Bays and Estuaries | 556.39 | 0 | not vulnerable |
| Lava | 539.18 | 0 | not vulnerable |
| Cliffs and Canyons | 474.44 | 0 | not vulnerable |
| Urban (High Intensity Developed) | 265.61 | 0 | addressed by “Buildings and People” HVRA values |
| Saltmarsh | 104.32 | 0 | not vulnerable |
| Rocky Coast | 3.67 | 0 | not vulnerable |
