# Supplementary material for: A quantitative wildfire risk assessment using a modular approach of geostatistical clustering and regionally distinct valuations of assets—A case study in Oregon
Source: PLoS One. 2022 Mar 8;17(3):e0264826. doi: 10.1371/journal.pone.0264826 (PMC8903305; doi:10.1371/journal.pone.0264826)
Supplement: S3 Table — Response function percentage values for FSA-specific risk assessments. (DOCX) [file pone.0264826.s007.docx]

**S3 Table Response function values used for overall relative risk calculation**

Table S6: Response function percentage values for FSA-specific risk assessments.

| **HVRA** | **Vulnerability class** | **Flame length class**  **1 2 3 4 5 6** | | | | | |
| --- | --- | --- | --- | --- | --- | --- | --- |
| Agriculture | 0 | 0 | 0 | 0 | 0 | 0 | 0 |
|  | 1 | -10 | -20 | -20 | -30 | -30 | -30 |
|  | 2 | -20 | -30 | -30 | -40 | -40 | -40 |
|  | 3 | -30 | -40 | -40 | -50 | -50 | -50 |
| WUI | - | -27 | -40 | -53 | -70 | -72 | -73 |
| Timber volume east  (FSA 3, 5, 6) | 0 | 0 | 0 | 0 | 0 | 0 | 0 |
|  | 1 | 30 | 20 | 15 | 10 | 10 | 0 |
|  | 2 | 20 | 15 | 10 | 5 | 5 | 0 |
|  | 3 | 0 | -2 | -4 | -6 | -8 | -10 |
|  | 4 | -10 | -15 | -20 | -25 | -25 | -30 |
|  | 5 | -30 | -35 | -40 | -45 | -45 | -50 |
|  | 6 | -50 | -55 | -60 | -65 | -65 | -70 |
|  | 7 | -70 | -75 | -80 | -85 | -85 | -90 |
|  | 8 | -90 | -95 | -95 | -95 | -95 | -95 |
|  | 9 | -95 | -95 | -95 | -95 | -100 | -100 |
| Timber volume west  (FSA 1, 2, 4) | 0 | 0 | 0 | 0 | 0 | 0 | 0 |
|  | 1 | -15 | -20 | -20 | -25 | -25 | -25 |
|  | 2 | -25 | -30 | -30 | -35 | -35 | -35 |
|  | 3 | -35 | -40 | -40 | -45 | -45 | -45 |
|  | 4 | -45 | -50 | -50 | -55 | -55 | -55 |
|  | 5 | -55 | -60 | -60 | -65 | -65 | -65 |
|  | 6 | -65 | -70 | -70 | -75 | -75 | -75 |
|  | 7 | -75 | -80 | -80 | -85 | -85 | -85 |
|  | 8 | -85 | -90 | -90 | -95 | -95 | -95 |
|  | 9 | -95 | -95 | -95 | -100 | -100 | -100 |
| Population density | 0 | 0 | 0 | 0 | 0 | 0 | 0 |
|  | 1 | -15 | -20 | -20 | -25 | -25 | -25 |
|  | 2 | -25 | -30 | -30 | -35 | -35 | -35 |
|  | 3 | -35 | -40 | -40 | -45 | -45 | -45 |
|  | 4 | -45 | -50 | -50 | -55 | -55 | -55 |
|  | 5 | -55 | -60 | -60 | -65 | -65 | -65 |
|  | 6 | -65 | -70 | -70 | -75 | -75 | -75 |
|  | 7 | -75 | -80 | -80 | -85 | -85 | -85 |
|  | 8 | -85 | -90 | -90 | -95 | -95 | -95 |
|  | 9 | -95 | -95 | -95 | -100 | -100 | -100 |
| Building density |  |  |  |  |  |  |  |
|  | 0 | 0 | 0 | 0 | 0 | 0 | 0 |
|  | 1 | -10 | -15 | -15 | -15 | -20 | -20 |
|  | 2 | -20 | -25 | -25 | -25 | -30 | -30 |
|  | 3 | -30 | -35 | -35 | -35 | -40 | -40 |
|  | 4 | -40 | -45 | -45 | -45 | -50 | -50 |
|  | 5 | -50 | -55 | -55 | -55 | -60 | -60 |
|  | 6 | -60 | -65 | -65 | -65 | -70 | -70 |
|  | 7 | -70 | -75 | -75 | -75 | -80 | -80 |
|  | 8 | -80 | -85 | -85 | -85 | -90 | -90 |
|  | 9 | -90 | -95 | -95 | -95 | -100 | -100 |
| Habitat | 0 | 0 | 0 | 0 | 0 | 0 | 0 |
|  | 1 | 0 | -10 | -10 | -20 | -20 | -20 |
|  | 2 | 0 | -5 | -15 | -25 | -25 | -25 |
|  | 3 | -5 | -10 | -20 | -30 | -30 | -30 |
|  | 4 | -5 | -15 | -25 | -35 | -35 | -35 |
|  | 5 | -10 | -20 | -30 | -40 | -40 | -40 |
|  | 6 | -15 | -25 | -35 | -45 | -45 | -45 |
|  | 7 | -20 | -30 | -40 | -50 | -50 | -50 |
|  | 8 | Not assigned in study area | | | | | |
|  | 9 | -30 | -40 | -50 | -60 | -60 | -60 |
| Drinking water (groundwater) | - | -5 | -10 | -10 | -20 | -20 | -20 |
| Drinking water (surface) | - | -10 | -20 | -40 | -60 | -60 | -60 |
